# Synthesis, Characterization, Photoluminescence, Molecular Docking and Bioactivity of Zinc (II) Compounds Based on Different Substituents

**DOI:** 10.3390/molecules25153459

**Published:** 2020-07-29

**Authors:** Rongping Liu, Hao Yan, Jinzhang Jiang, Jiahe Li, Xing Liang, Dengfeng Yang, Lixia Pan, Tisan Xie, Zhen Ma

**Affiliations:** 1 School of Chemistry and Chemical Engineering, Guangxi University, Nanning 530004, Guangxi, China; 18877571207@163.com (R.L.); jjz19941028@gmail.com (J.J.); lijiahe006@163.com (J.L.); liangxing587@126.com (X.L.); 2 National Engineering Research Center for Non-Food Biorefinery, State Key Laboratory of Non-Food Biomass and Enzyme Technology, Guangxi Academy of Sciences, Nanning 530004, Guangxi, China; 3 School of Animal Science and Technology, Guangxi University, Nanning 530004, Guangxi, China; yanhao_day@163.com; 4 Guangxi Key Laboratory of Marine Natural Products and Combinatorial Biosynthesis Chemistry, Guangxi Beibu Gulf Marine Research Center, Guangxi Academy of Sciences, Nanning 530004, Guangxi, China; dengfengyang@163.com

**Keywords:** zinc (II) complexes, antiproliferative activity, CT-DNA binding, fluorescence quenching, molecular docking

## Abstract

Six new zinc(II) complexes were prepared by the reaction of ZnBr_2_ or ZnI_2_ with 4′-(substituted-phenyl)-2,2′:6′,2′′-terpyridine compounds, bearing *p*-methylsulfonyl (L^1^), *p*-methoxy (L^2^) and *p*-methyl (L^3^), which were characterized by elemental analysis, FT-IR, NMR and single crystal X-ray diffraction. The antiproliferative properties against Eca-109, A549 and Bel-7402 cell lines and the cytotoxicity test on RAW-264.7 of these compounds were monitored using a CCK-8 assay, and the studies indicate that the complexes show higher antiproliferative activities than cisplatin. The interactions of these complexes with CT-DNA and proteins (BSA) were studied by UV-Vis, circular dichroism (CD) and fluorescent spectroscopy, respectively. The results indicate that the interaction of these zinc(II) complexes with CT-DNA is achieved through intercalative binding, and their strong binding affinity to BSA is fulfilled through a static quenching mechanism. The simulation of the complexes with the CT-DNA fragment and BSA was studied by using molecular docking software. It further validates that the complexes interact with DNA through intercalative binding mode and that they have a strong interaction with BSA.

## 1. Introduction

Cancer is one of the leading causes of death worldwide, with more than 10 million people suffering from the disease yearly [1,2,3]. Since its introduction in medical practice, cisplatin has been one of the most effective drugs for the treatment of cancers [4,5,6,7]. The biological activity of cisplatin comes from the fact that its covalent binding with DNA leads to cell death or apoptosis. However, there are also serious side effects such as nephrotoxicity, neurotoxicity, gastrointestinal toxicity, bone marrow suppression and drug resistance [8,9,10], among others. These side effects limit the application of platinum drugs and promote the development of alternative therapeutics [11,12,13]. The production of novel anticarcinogen medicines, which are less toxic, are more effective and target specific but show high efficacy in chemotherapy, is an active area of research [14,15]. The transition metal complexes have become candidate molecules for cancer treatment [16,17,18,19], and Zn(II) is of interest due to endogenous compatibility with the living system. Zinc is the second most abundant essential trace element in the human body, being involved in the synthesis of DNA and proteins [20,21]. Substituted terpyridine compounds are effective ligands for transition metal complexes [22,23,24,25]. Therefore, Zn(II) terpyridine complexes are worth exploring in molecular biology and related fields.

DNA and protein are two important biological targets in the research of anticancer drugs [26,27,28]. Studies have shown that one of the reasons for the cytotoxicity of cisplatin is its perfect non-specific combination with serum protein [29,30]. Therefore, the interaction between complexes and proteins plays a key role in the distribution and transport of small molecules into the biological system, which affects the concentration and overall efficacy of drug molecules [31]. In our previous studies, it has been found that zinc complexes have strong antitumor activity [22,23,32,33,34]. It is noteworthy that the antitumor properties of these complexes can be regulated by the substituents at their 4′-position. Hence, the exploration of the biological activity of different substituted zinc terpyridine complexes has a biological value for the design of the drug molecules.

**Scheme 1:**
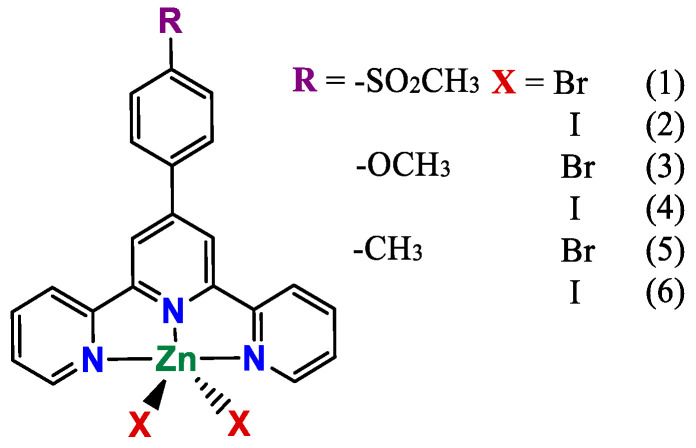
The compounds used in the experiment.

Six new zinc complexes **1**–**6** of substituted terpyridine compounds at the 4′-position with three chemical groups, *p*-methylsulfonyl (L^1^), *p*-methoxy (L^2^) and *p*-methyl (L^3^), were synthesized and characterized (Scheme 1). Their biological properties were studied as an extension of our previous work. The antiproliferative properties against Eca-109, A549 and Bel-7402 cell lines and the cytotoxicity test on RAW-264.7 of these compounds in vitro were studied using a CCK-8 assay. The interactions between the complexes with CT-DNA and a protein (BSA) were investigated by UV-Vis titration and CD spectroscopy (for the DNA) and fluorescence titration (for the protein). The binding constants (*K*_b_) and quenching constants (*K_q_*) for the interaction between these complexes and CT-DNA and BSA were determined. In addition, molecular docking was also carried out to verify their binding properties.

## 2. Results and Discussion

### 2.1. Syntheses and Characterization

**Figure 1:**
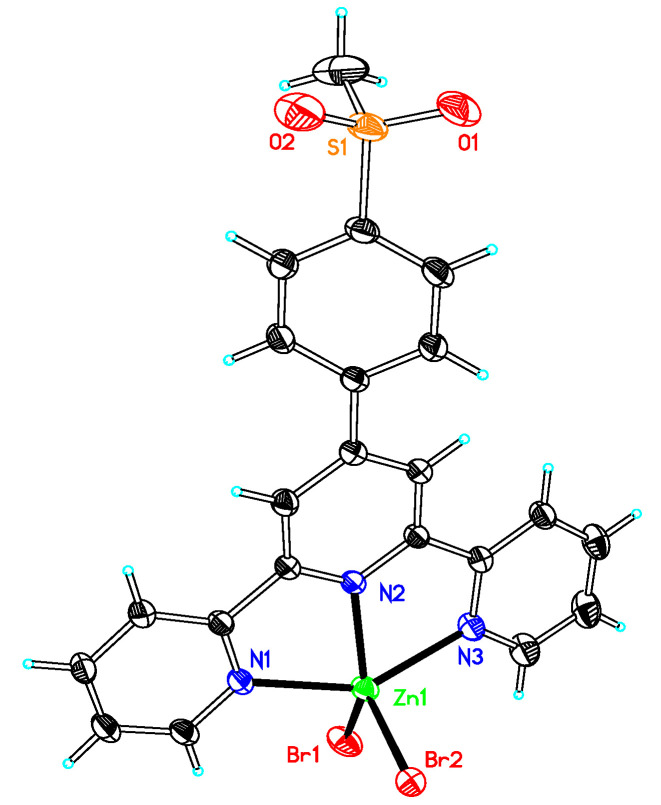
Thermal ellipsoid plot, drawn at the 50% probability level, of [ZnBr_2_L^1^] (1) with atomic numbering scheme. Selected bond lengths (Å) and angles (°): Zn(1)-N(2) 2.099(2), Zn(1)-N(1) 2.187(2), Zn(1)-N(3) 2.215(2), Zn(1)-Br(2) 2.4065(4), Zn(1)-Br(1) 2.4143(4), N(2)-Zn(1)-N(1) 74.22(8) N(2)-Zn(1)-N(3) 74.27(8), N(1)-Zn(1)-N(3) 146.24(8), N(2)-Zn(1)-Br(2) 138.77(6), N(1)-Zn(1)-Br(2) 98.90(6) N(3)-Zn(1)-Br(2) 96.78(5), N(2)-Zn(1)-Br(1) 108.69(6), N(1)-Zn(1)-Br(1) 97.22(6) N(3)-Zn(1)-Br(1) 104.06(6), Br(2)-Zn(1)-Br(1) 112.512(17).

Ligands L^1^–L^3^ were prepared according to reported procedures [23,33]. All the complexes were synthesized by reaction of the ligands with ZnBr_2_ or ZnI_2_ in stoichiometric amounts, respectively. Complexes **1**–**6** were characterized by ^1^H-NMR spectroscopy (Figures S1–S6, Supplementary Materials), IR (Figures S7–S12), elemental analysis and single crystal X-ray diffraction (Figure 1 and Figure S13–S17). IR spectra of all the compounds show the pyridyl and phenyl characteristic bands. Compounds **1** and **2** appear at the methylsulfonyl characteristic band at 1305 cm^−1^ and 1145 cm^−1^. The bands at 1235 cm^−1^ and 1184 cm^−1^ belong to the absorption of methoxy (**3** and **4**) and those at 1380 cm^−1^ and 1442 cm^−1^ belong to the absorption of methyl (**5** and **6**). As shown in Figures S1–S6 (Supplementary Materials), the hydrogen atoms at the pyridyl rings of all the iodide complexes (**2**, **4** and **6**) are affected by the spatial position of the iodine atom, leading to the different chemical environments of the hydrogen atoms and the different chemical shift values in the nuclear magnetic spectrum. It is interesting that the maximum and minimum chemical shifts of the pyridyl hydrogens decrease accordingly with the substituent groups’ sequence of methylsulfonyl (–SO2CH3)→methoxy (–OCH3)→methyl (–CH3).

### 2.2. Elucidation of the Structures of the Compounds

Crystals of all the complexes were obtained by evaporation of their DMF solutions, which were suitable for X-ray analysis. 

Compound **1** is a mononuclear complex that crystallized in a centrosymmetric space group *C*2*/c* with one molecule per asymmetric unit, as shown in Figure 1. The zinc ion is coordinated by three *N* atoms of the 4′′-methylsulfonyl-4′-phenyl-2,2′:6′,2′′-terpyridine ligand and two bromide anions. In order to better understand the geometry of the complex, its value for the tau parameter was calculated as 0.56 [35]. The result shows that its coordination environment is distorted trigonal-bipyramidal. The average bond length of the Zn–N bond is 2.167 Å, and that of the Zn–Br bond is 2.4104 Å. The crystal structure shows that the three pyridine groups are basically planar with the root-mean-square (RMS) deviation being 0.1267 Å. The phenyl with methylsulfonyl substituent forms a dihedral angle between the central pyridine ring of 26.79(9)° and the plane determined by Zn–N1–N2–N3 of 25.22(10)° Å. There are no classical hydrogen bonds in the structure but several hydrogen bonds between the hydrogen of carbon atoms (C1, C18, C20 and C22) and neighboring atoms (O atom and Br atom) in the structure. The distance for the hydrogen bonds of C1–H…O1 is 3.2878(1) Å, and it is 2.8903(1) for C18–H…O2, 3.6487(1) for C18–H…Br2 and 3.8044(1) Å for C22–H…Br1. An intermolecular π–π interaction is detected, which relates to a middle pyridyl and a plane defined by Zn1–N2–C10–C11–N3, with a centroid distance of 3.6348(1) Å. There is a π–ring interaction in the form of Y–X…Cg in the structure with the distance (X…Cg) of 3.9082(1) Å, involved in the oxygen atom at the phenyl group and the middle pyridine ring of the neighboring ligand.

Like **1**, **2**–**6** are also mononuclear species crystallized in space groups, i.e., *P*2_1_2_1_2_1_ for **2**, *P*2_1_*/c* for **3**, **5** and **6** and *P*2_1_/*n* for **4** (Figures S13–S17, Supplementary Materials). The selected bond angles and lengths of **2**–**6** are listed in the captions of Figures S13–S17. In order to better understand the geometry of the complexes, the values of the *tau* parameter were also calculated (τ = 0.48, 0.60, 0.54, 0.56 and 0.59 for compounds **2**–**6**, respectively) [35]. The results show that the coordination environment for compounds **3**–**6** is distorted trigonal-bipyramidal, whereas it is a distorted square-pyramidal for compound **2**. All the complexes present a π–ring interaction in the structures. In **2**, there are two π–ring (Y–X…Cg) interactions with atom-centroid distances (X…Cg) being 3.9525(7) Å and (Y…Cg) being 4.7005(8) Å. In **3**, **4**, **5** and **6**, there are two kinds of π–ring (X–H…Cg) interactions, giving the atom-centroid distance (X…Cg) of 3.9183(1) for **3**, 3.7288(1) for **4**, 3.7543(1) and 3.7348(1) for **5** and 3.814(5) Å for **6** as well as (H…Cg) of 2.960 for **3**, 2.990 for **4**, 2.910 and 2.820 for **5** and 2.870 Å for **6**, respectively. In **4**, there is one kind of π–π interaction, showing a distance of 3.5953(1) Å between one terminal pyridyl N1–C1–C2–C3–C4–C5 and one plane defined by Zn1–N1–C5–C6–N2.

### 2.3. Photoluminescent Properties

**Figure 2:**
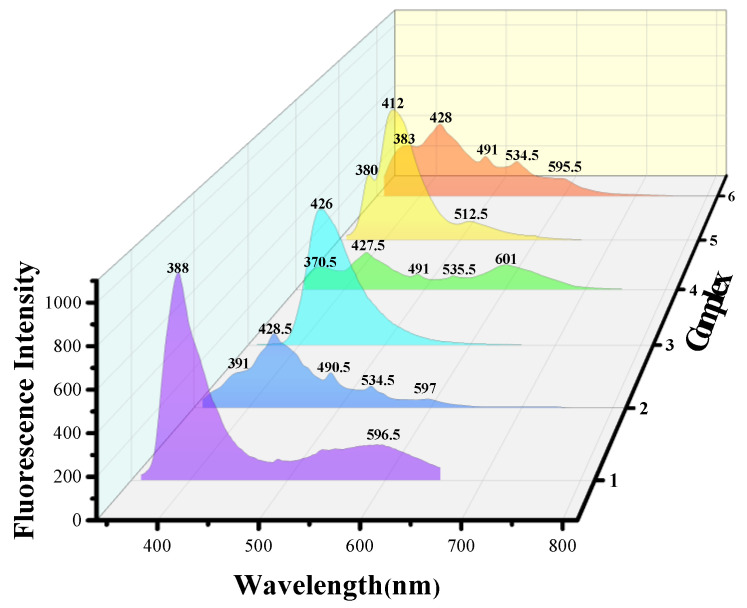
Solid state emission spectra (excitation at 300 nm) of complexes **1**–**6** at room temperature.

The emission spectra of all the compounds are shown in Figure 2. Due to the difference between the substituted groups and the anionic ligands, the solid photoluminescent properties of these complexes at room temperature are interesting. The zinc bromide complexes **1**, **3** and **5** have only one strong peak (388 nm for **1**, 426 nm for **3** and 412 nm for **5**). As the substituent groups vary from electron absorption to donor electron (**1**→**5**→**3**), the emission spectra were redshifted. Compounds **1** and **5** show one or two low strength bands (596.5 nm for **1**, 380 nm and 512.5 nm for **5**). However, zinc iodide complexes **2**, **4** and **6** show five bands in the emission spectra and there is an obvious strength band at 428.5 nm, 427.5 nm and 428 nm, respectively. Four other low strength bands show their positions at 391 nm, 490.5 nm, 534.5 nm and 597 nm for **2**, 370.5 nm, 491 nm, 535.5 nm and 601 nm for **4** and 383 nm, 491 nm, 534.5 nm and 595.5 nm for **6**. It is interesting that the strong peaks of the zinc iodide compounds do not change visibly and that the peaks have redshifted in comparison with the strong peaks of the zinc bromide complexes. According to the reported photoluminescent results of 4′-phenyl-terpyridine and other substituted terpyridine compounds, the peaks around 380 nm are assigned tentatively to the π–π*/MLCT mechanism [22,23]. The other peaks of the iodide anion compounds (**2**, **4** and **6**) in the low-energy region are assigned temporarily to *n*–π*/LMCT [22].

### 2.4. Solution Stability

**Figure 3:**
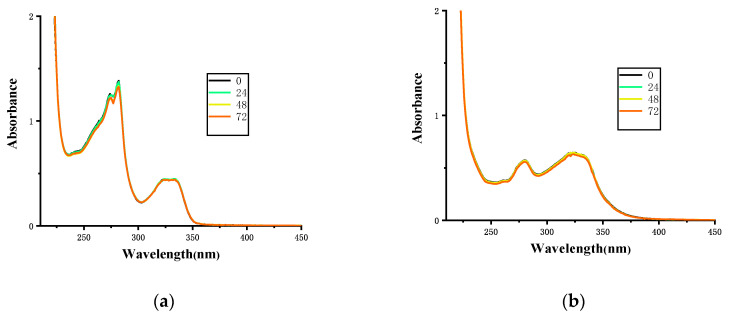
The UV-vis spectra of complexes (**a**) **1** and (**b**) **3** in PBS buffer solution over a period of 72 h.

The stability of complexes **1**–**6** under physiological conditions (PBS buffer, pH = 7.4) was monitored by UV-Vis spectroscopy. Stock concentrations of complexes **1**–**6** were prepared in DMSO and diluted with PBS buffer solution. The stability analysis was performed at 0, 24, 48 and 72 h. As shown in Figure 3 for **1** and **3** (**2**, **4**, **5** and **6** are shown in Figure S18, Supplementary Materials), there are no obvious changes in the UV-Vis spectra of the compounds, indicating that the complexes are stable under physiological conditions.

### 2.5. Antiproliferative Activity and Toxicity Studies

**Figure 4:**
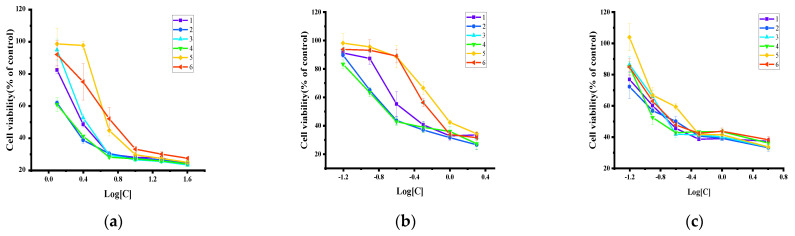
The curves of antitumor activities of compounds **1**–**6** against (**a**) Eca-109, (**b**) A549 and (**c**) Bel-7402 cell line.

The in vitro antiproliferative properties of complexes **1**–**6** against Eca-109, A549 and Bel-7402 tumor lines were examined by employing the CCK-8 assay with cisplatin as a positive control. As seen from Figure 4, all the complexes display a dose-dependent manner on the viability of the test cells. Table 1 shows the IC_50_ values of the compounds in the range of 0.066–0.945 μM, which is significantly lower than that of cisplatin (5.66–11.45 μM). It is interesting that the different cells respond distinctively to these complexes. The complexes display remarkable cytotoxicity against Eca-109 in comparison to A549 and Bel-7402. Notably, the antiproliferative activity of **1** and **2** is better than that of the other compounds towards the three cell lines and, when the para-substituent is the same, the activity of the iodide anion complexes is generally better than that of the bromide anion ones. Compared with that of zinc chloride complexes [34], the antiproliferative activity of these complexes is stronger. This activity of the bromide complexes follows the order of **1** > **3** > **5** and that of the iodide complexes is **2** > **4** > **6**. These results reveal that both the substituents and counterion have effects on the activity. It is worth mentioning that, among these compounds, the order of the activity is consistent with the electronegativity values (methylsulfonyl (−2.96) < methoxy (−2.86) < methyl (−2.28) of the substituents. 

**Figure 5:**
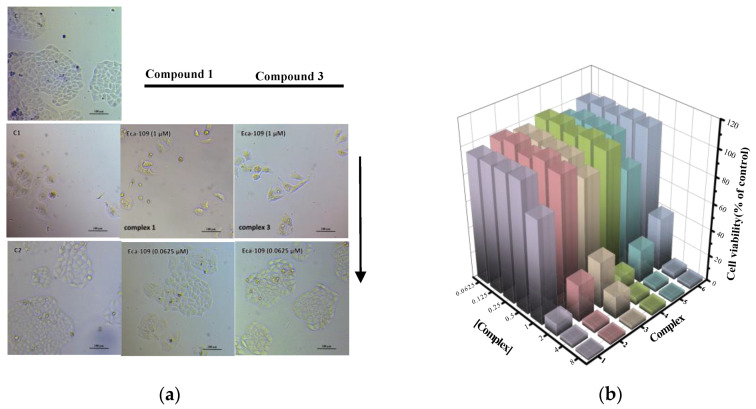
(**a**) Microscopic photographs of Eca-109 cancer cells treated with different concentrations of compound **1** and control photographs (C for blank control group, C1 for 2.5 μM cisplatin, C2 for 20 μM cisplatin). (**b**) The cell viability of compounds **1–6** against RAW264.7.

The viability of RAW264.7 cells treated with compounds **1**–**6** was tested following the same methods used on the tumor cell lines, and the plots of cell viability of compounds **1**–**6** in increasing concentration are shown in Figure 5b. The results reveal that the compounds promote the growth of RAW264.7 cells at low concentrations (<1 µM) and inhibit the growth of the cells at high concentrations (>1 µM). As seen by the CCK-8 results, complexes **1**–**6** show significant cytotoxicity at 0.066–0.945 μM towards the cancer cells with no apparent cytotoxicity towards the normal cells.

### 2.6. Protein Binding Analysis

#### 2.6.1. Fluorescence Quenching Analysis

**Figure 6:**
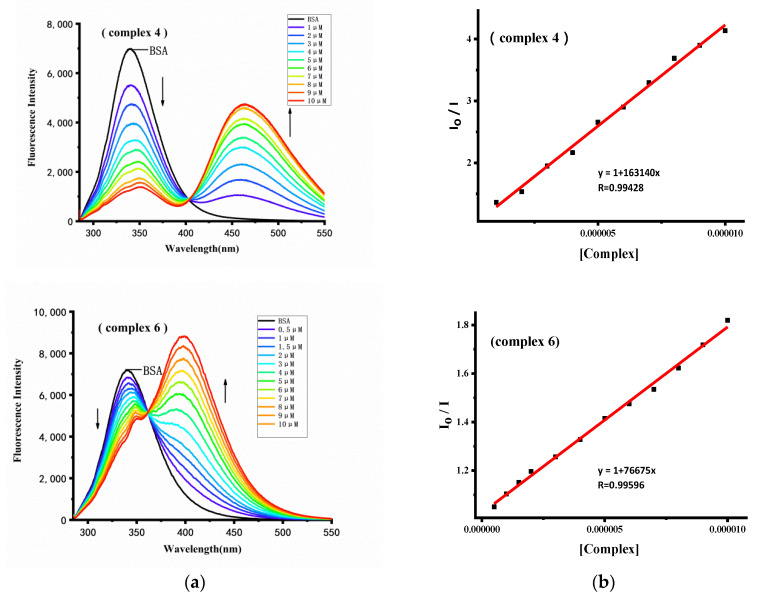
(**a**) Fluorescence emission spectra of 1.6 μM BSA in a Tris-HCl buffer (pH = 7.2) solution with series concentration of compounds **4** and **6**, and (**b**) curves for the Stern–Volmer equation as the insets.

The structure and transmission characteristics of BSA are similar to HSA [36]. BSA was used instead of HSA for drug delivery due to its ease to purify and stability. The binding of metal complexes to proteins was studied by fluorescence spectrum titration. Endogenous fluorescence of BSA comes from residues as tryptophan (Trp), tyrosine (Tyr) and phenylalanine (Phe), and conformational changes of BSA-binding metal complexes are obtained by observing the fluorescence of Trp residues at 350 nm [27]. Figure 6 shows the fluorescence emission spectra of BSA (1.6 μM) incubated with different concentrations of complexes **4** (0–20 μM) and **6** (0–20 μM). Interestingly, with the increase of concentration of **4** at 350 nm, BSA shows strong fluorescence quenching, accompanied by fluorescence enhancement at 450 nm. The same phenomenon occurs for the other compounds (shown in Figure S22, Supplementary Materials). It is worth mentioning that, unlike other complexes, low concentration of **1** and **2** significantly enhances fluorescence intensity at 380 nm when added into BSA, while the quenching is relatively weak. This appearance may be due to a resonant energy transfer. The strong quenching effect indicates that the metal complexes are bound to BSA and change the microenvironment around Trp residues. The Stern–Volmer equation [37] (Equation (1)) was used to study the quenching mechanism of BSA and the complexes:

*I*0*/I* = 1 + *K_q_*τ0[*Q*] = 1 + *Ksv*[*Q*] (1)

where the fluorescence intensity of the protein is expressed by *I*0 and I in absence and in presence of quencher, respectively; *Ksv* is the Stern–Volmer quenching rate constant; *K_q_* is the quenching rate constant; and *τ*0 and [*Q*] represent the lifetime of fluorescent molecule in absence of quencher (valued 10^−8^ s) and quencher concentration, respectively. The maximum causing a value of dynamic quenching in biopolymers is 2 × 10^10^ M^−1^ S^−1^ [23]. In Table 2, it can be observed that the range of *K_q_* is 4.6 × 10^12^ to 1.8 × 10^13^, indicating that the interaction of the complexes and BSA is a static quenching mechanism.

#### 2.6.2. Binding Constant and Site Analysis

**Figure 7:**
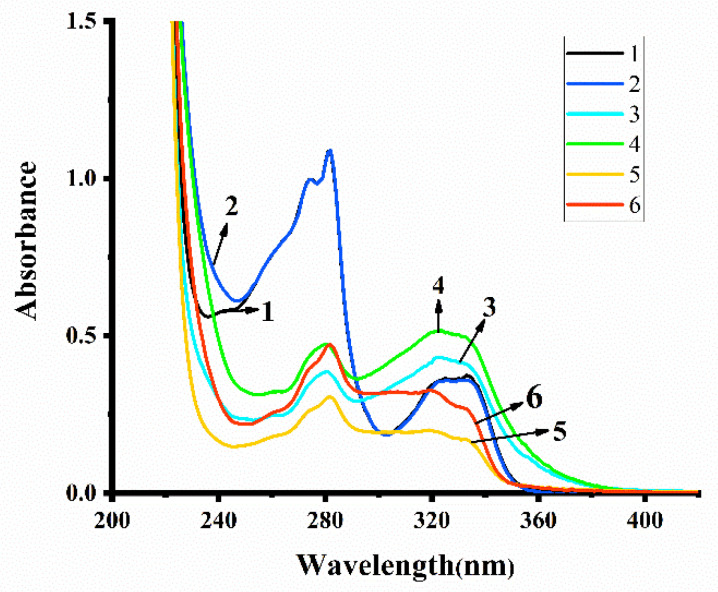
Absorption spectra of 20 μM of compounds **1**–**6** in Tris-HCl buffer.

Calculation of the binding constants (*K_b_*) was achieved according to the Scatchard equation [38] (Equation (2)):

log [(*I*0 − *I*)/*I*] = log*K_b_* + *n*log[*Q*] (2)

where *K_b_* and *n* represent the binding constant and the binding site number, respectively. The binding constants *K_b_* and the binding site number were obtained from the intercept and slope of the double logarithm regression plot between log [(*I*_0_ − *I*)/*I*] and log [*Q*]. The data are shown in Table 2. The binding constant (>104 L mol^−1^) was obtained under the condition of high binding affinity [39]. The *K_b_* range is 4.5 × 10^3^ to 3.8 × 10^5^ L∙mol^−1^ and the binding site number is equivalent to 1. The experimental results show that complexes **1**–**6** can effectively bind to BSA, affecting the microenvironment of Trp residues and bringing drugs into the target cells to promote apoptosis [40,41,42]. It can be seen in Figure 7 that the absorption spectra of the complexes partially overlap with the excitation spectrum of BSA. 

#### 2.6.3. Protein Docking

**Figure 8:**
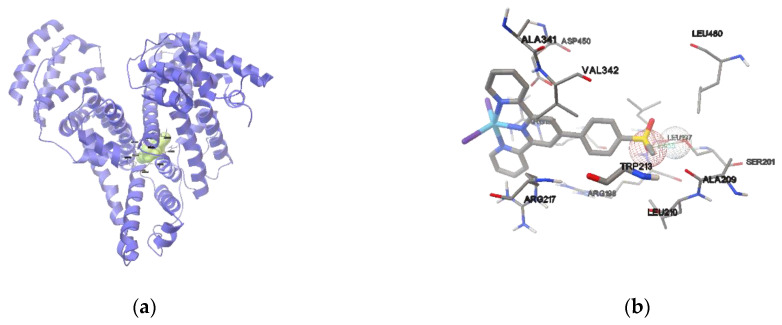
Docking conformation of complex **1** to (**a**) BSA and (**b**) amino acid residues surrounding **1**.

Molecular docking is helpful to know the interaction of complexes and BSA [43,44]. As shown in Figure 8 for **1** (Figure S23, Supplementary Materials, for the other compounds), it enters and is embedded in the hydrophobic cavity of subdomain IIA of BSA at binding site I [45,46,47]. The complex interacts with BSA protein primarily through a hydrogen bond and van der Waals force. It is seen that **1** is surrounded by amino acid residues such as TRP-213, ARG-194, AlA-341, AlA-209, ASP-450, VAL-342, ARG-217, LEU-480, SER-201, and LEU-210. The binding energies for all the compounds are listed in Table 3, namely −7.69 (**1**), −8.28 (**2**), −7.00 (**3**), −6.99 (**4**), −7.26 (**5**) and −7.79 (**6**). The results show that the other complexes can also spontaneously enter into the hydrophobic region of the subdomain IIA of BSA like **1**. 

### 2.7. DNA Binding Studies

#### 2.7.1. UV-Vis Spectroscopy

UV-Vis titration absorption spectrometry is one of the effective methods to study the binding pattern of metal complexes to DNA [48,49]. When the complexes are inserted into the DNA base interlayer and the aromatic group of the complexes and the DNA base generate a π–π interaction, the absorption spectrum shows hypochromism and redshift [50,51]. While the complexes are inserted into the DNA groove, the absorption band (LMCT) shows hyperchromicity and blueshift [52]. The binding pattern was determined by the change observed in the absorption spectrum. The binding affinity was evaluated by calculating the binding constant (*K_b_*). The equation [27] (Equation (3)) is as follows:

A_0_/A − A_0_ = 1/*f_a_* + 1/*f_a_K_b_*[DNA] (3)

**Figure 9:**
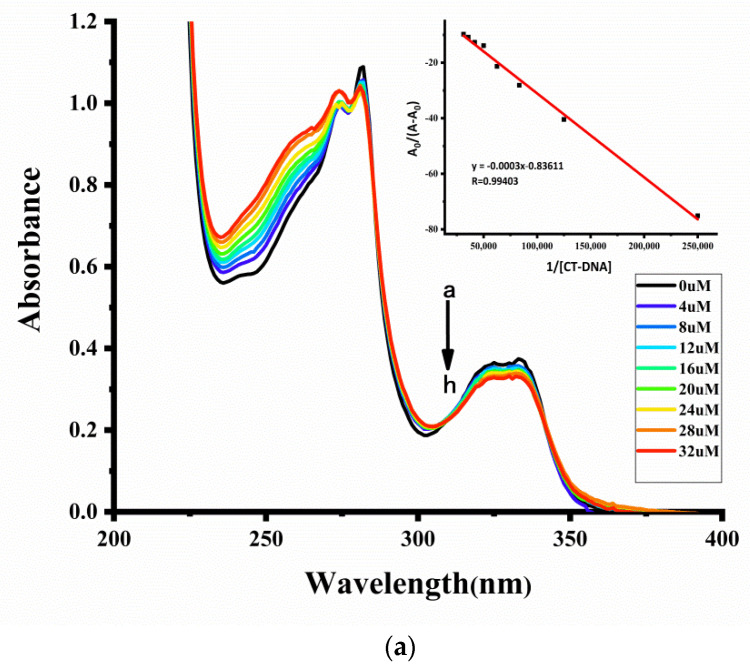

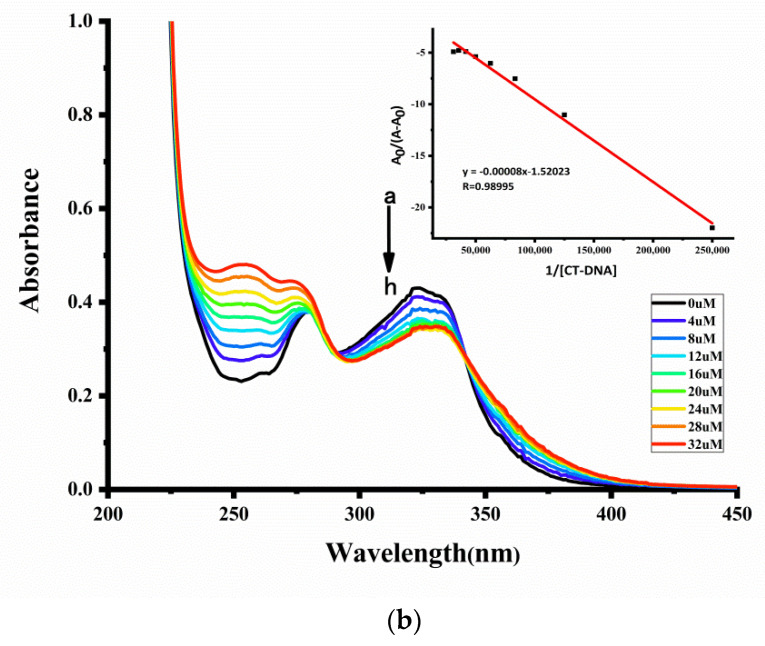
Absorption spectra of 20 μM of compounds (**a**) **1** and (**b**) **3** in a Tris-HCl buffer (pH = 7.2) solution with series concentrations of CT-DNA. The plots of A_0_/(A − A_0_) versus the concentration of CT-DNA are shown as the insets.

The absorption spectra of the metal complexes and CT-DNA are shown in Figure 9 (**1** and **3**) and Figure S24, Supplementary Materials (the other compounds). With an increase of CT-DNA concentrations, the LMCT absorption bands of the complexes give the results of a significant hypochromism, which shows that the interaction between the complexes and DNA is consistent with the embedding mode. This mode is confirmed by molecular docking calculation and CD spectroscopy. The binding constants (*K_b_*) of the metal complexes and CT-DNA are shown in Table 4, indicating that all the metal complexes have strong interactions with DNA. The results show that the binding constants of the compounds with DNA have a tendency of increase, which has a relationship with the 4-position substituents of the compounds to give a spatial effect that the binding constants increase as the electronegativity of the substituents decreases. This tendency is also contrary to the order of the antiproliferative ability of these compounds against the cells.

#### 2.7.2. Circular Dichroism Analysis

**Figure 10:**
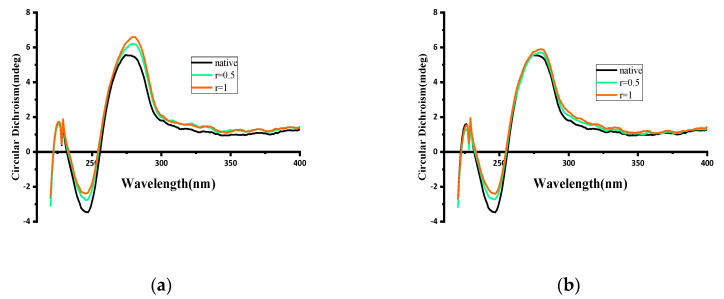
CD spectra of compounds (**a**) **1** and (**b**) **3** to CT-DNA at different concentration ratios.

CD spectroscopy was further used to study the DNA interacting behavior with complexes **1**–**6**. CD study is an effective method to detect minor conformational change of DNA induced by metal drug binding. The characteristic CD spectra of B-form of CT-DNA (calf thymus DNA) displays a positive band at 275 nm (due to base stacking) and a negative band at 246 nm (related to right-handed helicity) due to the coupling of the stacked planar bases arranged in chiral helices [53,54]. Simple groove binding does not cause observable changes in the positive band at 275 nm, whereas intercalation impacts the intensities of the negative and positive bands. As Figure 10 and Figure S25 (Supplementary Materials) show, the CD spectrum of CT-DNA with different ratios of the complexes was detected in Tris-HCl buffer solution systems. It can be clearly seen that obvious changes are observed with the participation of the complexes in both positive and negative bands. The intensity of the negative bands decreases and that of the positive bands increases. The results indicate that the metal complexes with DNA display an intercalation binding mode, which is consistent with the conclusion of the UV-Vis titration study. The binding effect of the bromine compounds with DNA has a tendency of **1** > **5** > **3,** which may be determined by both electronegativity and spatial effect. However, differing from that of the bromide complexes, the binding effect of the iodide compounds with DNA increases and this consequence has a relationship with the effects of the 4-position substituents of the compounds, which displays a spatial effect on the binding of the compounds with DNA. 

#### 2.7.3. Molecular Docking Studies with CT-DNA

**Figure 11:**
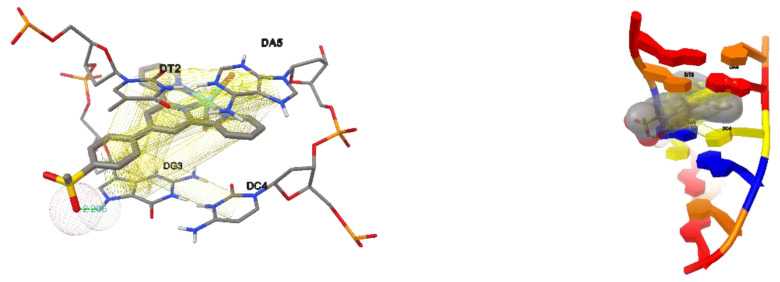
Docking conformation of complex **1** to DNA.

Molecular docking technology is an important tool to know the interaction between small molecules of drugs and biological targets in medicinal research [55,56,57]. The molecular docking study was carried out by the structure of the zinc (II) complexes with DNA, and the modes and energy of the binding were calculated. As shown in Figure 11 and Figure S26 (Supplementary Materials), all the complexes are bound primarily by intercalative mode in DNA. The small molecule inserts at the base interlayer, to produce a π–π interaction with the base. In addition, the binding energy of complexes 1–6, as shown in Table 3, is in the range of −7.90 to −8.78 kcal mol^−1^. It is clear from the binding energies that the intercalation of the compounds into DNA is spontaneous and effective. The results show that the binding energy of the compounds with DNA has a tendency to decrease when the anionic ligands are the same, which is related to the 4-position substituents of the compounds. This consequence is different from the order of the antiproliferative ability of these compounds against the cancer cells. 

## 3. Materials and Methods

^1^H-NMR spectra were determined with an Agilent Technologies 800/54 premium (Rogowska, Wroclaw, Poland). Elemental analyses were run on an Elementar Vario EL III Elemental Analyzer (Langenselbold, Frankfurt, Germany). IR spectra were recorded on a Nicolet IS10 (Carlsbad, CA, USA). Intensity data of single crystals were collected using an Agilent SuperNova diffractometer (Santa Clara, CA, USA). UV-Vis absorption spectra were performed using a Beckman coulter DU800 (Brea, CA, USA). Fluorescence spectra were recorded on a PerkinElmer LS 55 (Waltham, MA, USA) luminescence spectrometer with a red-sensitive photomultiplier type R928. Circular dichroism (CD) spectra carried out on a Chirascan CD spectropolarimeter (Applied Photophysics, Leatherhead, Surrey, U.K.). All the cells were purchased from the American Type Culture Collection (ATCC, Manassas, VA, USA) and Tris-HCl buffer, BSA and CT-DNA were obtained from Beijing Solarbio Science & Technology Co., Ltd., Asbio and Solarbio (Beijing, China), respectively. BSA were dissolved in Tris-HCl buffer and stored at 4 °C. The complexes were dissolved in DMSO to prepare stock solution and the solubility of the complexes was 20 mM/mL in DMSO, while the solubility of **5** was 15 mM/mL. All reagents used in the experiments were of analytical grade or purified by standard methods. 

### 3.1. Synthesis

The ligands L^1^–L^3^ were prepared by following reported procedures [23,33]. Compounds **1**–**6** were synthesized by reaction of the ligands with zinc salts (ZnBr_2_ or ZnI_2_). A 10 mL methanol solution of zinc salt (9 × 10^−4^ mol) was slowly added into 10 mL dichloromethane solution of the ligands (9 × 10^−4^ mol). The mixture was stirred for 24 h at room temperature. The compounds were white (**1**, **2**, **5** and **6**), yellow (**3**) or orange (**4**) powders and characterized by elemental analysis, IR spectroscopy and NMR analysis. Suitable crystals for determination were obtained by slow evaporation of the solutions of the compounds in DMF in different colors.

#### 3.1.1. [ZnBr_2_L^1^] (**1**)

A white solid (yield: 80%) was obtained upon filtration of the solution and washed with CH_2_Cl_2_/CH_3_OH and dried in a desiccator. Orange crystals were obtained by evaporation of its DMF solution, which were suitable for X-ray analysis. Anal. calcd for C_22_H_17_Br_2_N_3_O_2_SZn: C, 43.13, H, 2.80, N, 6.86%. Found: C, 43.24, H, 2.78, N, 6.90%. ^1^H-NMR (800 MHz, DMSO-*d*_6_) δ 9.18 (s, 2H), 8.95 (d, *J* = 7.9 Hz, 2H), 8.89 (s, 2H), 8.49 (d, *J* = 7.9 Hz, 2H), 8.36 (t, *J* = 7.9 Hz, 2H), 8.19 (d, *J* = 7.9 Hz, 2H), 7.90 (d, *J* = 6.5 Hz, 2H), 3.35 (s, 4H). IR (cm^−1^): 3060 (m), 1615 (s), 1598 (s), 1576 (s, V_pyridyl-H_), 1548 (s), 1474 (vs, V_pyridyl-H_), 1427 (s), 1391 (s), 1305 (vs), 1251 (m), 1145 (vs), 1092 (s), 1012 (s), 951 (m), 837 (m), 791 (vs, γ_aryl-H_), 769 (s), 732 (s), 673 (s), 636 (m).

#### 3.1.2. [ZnI_2_L^1^] (**2**)

A white solid (yield: 62%) was obtained upon filtration of the solution and washed with CH_2_Cl_2_/CH_3_OH and dried in a desiccator. Brown crystals were obtained by evaporation of its DMF solution, which were suitable for X-ray analysis. Anal. calcd for C_22_H_17_I_2_N_3_O_2_SZn: C, 37.39, H, 2.42, N, 5.95%. Found: C, 37.52, H, 2.39, N, 5.97%. ^1^H-NMR (800 MHz, DMSO-*d*_6_) δ 9.46 (s, 1H), 9.21 (s, 1H), 9.15 (d, *J* = 8.0 Hz, 1H), 9.03 (d, *J* = 7.9 Hz, 1H), 8.96–8.93 (m, 1H), 8.66 (d, *J* = 7.9 Hz, 1H), 8.52 (d, *J* = 7.9 Hz, 1H), 8.43 (t, *J* = 7.9 Hz, 1H), 8.30 (t, *J* = 8.2 Hz, 2H), 8.21 (d, *J* = 7.9 Hz, 1H), 7.97 (dt, *J* = 24.2, 4.8 Hz, 2H), 7.51 (t, *J* = 6.2 Hz, 1H), 3.33 (d, *J* = 2.1 Hz, 3H). IR (cm^−1^): 3069 (m), 1613 (s, V_pyridyl-H_), 1599 (s), 1570 (m, V_pyridyl-H_), 1548 (s, V_pyridyl-H_), 1475 (vs, V_pyridyl-H_), 1427 (s), 1393 (s), 1313 (vs), 1248 (m), 1147 (vs), 1093 (s), 1012 (s), 960 (m), 832 (m), 792 (vs, γ_aryl-H_), 768 (s), 731 (s), 657 (s), 637 (s).

#### 3.1.3. [ZnBr_2_L^2^] (**3**)

A yellow solid (yield: 75%) was obtained upon filtration of the solution and washed with CH_2_Cl_2_/CH_3_OH and dried in a desiccator. Orange crystals were obtained by evaporation of its DMF solution, which were suitable for X-ray analysis. Anal. calcd for C_22_H_17_Br_2_N_3_OZn∙H_2_O: C, 45.35 H, 3.29, N, 7.21%. Found: C, 45.25, H, 3.32, N, 7.02%. ^1^H-NMR (800 MHz, DMSO-*d*_6_) δ 9.02 (s, 2H), 8.93 (d, *J* = 7.9 Hz, 2H), 8.86 (d, *J* = 4.9 Hz, 2H), 8.32 (t, *J* = 7.7 Hz, 2H), 8.26 (d, *J* = 8.3 Hz, 2H), 7.89–7.86 (m, 2H), 7.17 (d, *J* = 8.2 Hz, 2H), 3.87 (s, 3H). IR (cm^−1^): 3049 (m), 2838 (m), 1592 (vs), 1572 (s, V_pyridyl-H_), 1541 (m), 1519 (vs), 1476 (s, V_pyridyl-H_), 1433 (s), 1408 (s), 1235 (vs), 1184 (s), 1066 (s), 1018 (s), 837 (s), 789 (vs, γ_aryl-H_), 732 (s), 635 (s), 578 (s).

#### 3.1.4. [ZnI_2_L^2^] (**4**)

An orange solid (yield: 70%) was obtained upon filtration of the solution and washed with CH_2_Cl_2_/CH_3_OH and dried in a desiccator. Brown crystals were obtained by evaporation of its DMF solution, which were suitable for X-ray analysis. Anal. calcd for C_22_H_17_I_2_N_3_OZn: C, 40.12, H, 2.60, N, 6.38%. Found: C, 40.23, H, 2.07, N, 6.23%. ^1^H-NMR (800 MHz, DMSO-*d*_6_) δ 9.33 (s, 1H), 9.15 (d, *J* = 8.1 Hz, 1H), 9.07 (s, 1H), 9.03 (d, *J* = 7.9 Hz, 1H), 8.92 (d, *J* = 5.0 Hz, 1H), 8.46 (d, *J* = 8.1 Hz, 1H), 8.40 (t, *J* = 7.7 Hz, 1H), 8.31–8.25 (m, 2H), 7.93 (t, *J* = 5.6 Hz, 2H), 7.47 (dd, *J* = 7.3, 5.2 Hz, 1H), 7.28 (d, *J* = 8.2 Hz, 1H), 7.20 (d, *J* = 8.3 Hz, 1H), 3.91 (d, *J* = 40.6 Hz, 3H). IR (cm^−1^): 3063 (m), 2838 (m), 1596(s), 1567 (s, V_pyridyl-H_), 1541 (m), 1516 (s), 1472 (s, V_pyridyl-H_), 1431 (s), 1431 (s), 1403 (s), 1241 (s), 1184 (s), 1069 (m), 1009 (s), 876 (m), 826 (s), 790 (vs, γ_aryl-H_), 727(s), 731 (s), 638 (s), 578 (s).

#### 3.1.5. [ZnBr_2_L^3^] (**5**)

A white solid (yield: 73%) was obtained upon filtration of the solution and washed with CH_2_Cl_2_/CH_3_OH and dried in a desiccator. Orange crystals were obtained by evaporation of its DMF solution, which were suitable for X-ray analysis. Anal. calcd for C_22_H_17_Br_2_N_3_Zn: C, 48.16, H, 3.12, N, 7.66%. Found: C, 48.61, H, 2.74, N, 7.65%. ^1^H-NMR (800 MHz, DMSO-*d*_6_) δ 9.07 (s, 2H), 8.96 (d, *J* = 8.0 Hz, 2H), 8.87 (d, *J* = 4.8 Hz, 2H), 8.34 (t, *J* = 7.8 Hz, 2H), 8.19 (d, *J* = 7.7 Hz, 2H), 7.92–7.87 (m, 2H), 7.46 (d, *J* = 7.8 Hz, 2H), 2.43 (s, 3H). IR (cm^−1^): 3069 (m), 1611 (s), 1595 (s), 1573 (s, V_pyridyl-H_), 1545 (s), 1473(s, V_pyridyl-H_), 1425 (s), 1402 (s), 1251 (s), 1150 (m), 1012 (s), 879 (m), 829 (s), 792 (vs, γ_aryl-H_), 732 (s), 689 (s), 657 (s), 634 (s).

#### 3.1.6. [ZnI_2_L^3^] (**6**)

A white solid (yield: 61%) was obtained upon filtration of the solution and washed with CH_2_Cl_2_/CH_3_OH and dried in a desiccator. Brown crystals were obtained by evaporation of its DMF solution, which were suitable for X-ray analysis. Anal. calcd for C_22_H_17_I_2_N_3_Zn: C, 41.12, H, 2.66, N, 6.54%. Found: C, 41.15, H, 2.50, N, 6.60%. ^1^H-NMR (800 MHz, DMSO-*d*_6_) δ 9.36 (s, 1H), 9.15 (d, *J* = 8.1 Hz, 1H), 9.11 (s, 1H), 9.04 (d, *J* = 8.0 Hz, 1H), 8.92 (d, *J* = 4.9 Hz, 1H), 8.41 (d, *J* = 7.8 Hz, 1H), 8.37 (d, *J* = 7.6 Hz, 1H), 8.27 (d, *J* = 7.8 Hz, 1H), 8.20 (s, 1H), 7.94 (d, *J* = 4.3 Hz, 2H), 7.57 (s, 1H), 7.48 (d, *J* = 7.1 Hz, 2H), 2.43 (s, 3H). IR (cm^−1^): 3051 (m), 1609 (s), 1573 (s, V_pyridyl-H_), 1544 (s), 1473 (s, V_pyridyl-H_), 1424 (s), 1400 (s), 1254 (m), 1155 (s), 1012 (s), 888 (m), 824 (s), 791 (vs, γ_aryl-H_), 731 (s), 689 (s), 657 (s), 634 (s).

### 3.2. X-ray Determinations

Crystals of complexes **1**–**6** intensity data were collected using an Agilent SuperNova single crystal diffractometer (Santa Clara, CA, USA) with omega scans of 0.5° per frame with the monochromatic Mo Kα (λ = 0.71073 Å) radiation. Cell parameters were retrieved by Agilent CrysAlisPro (Santa Clara, CA, USA) [58], and all the reflections refinement were performed using Agilent CrysAlisPro [59]. The direct method was used to solve structures using the SHELXS-97 (Göttingen, Niedersachsen, Germany) [60] package and refined using SHELXL-97. Calculations were carried out by the WinGX System-Version 1.80.03 (Glasgow, Scotland, U.K.) [61]. The remaining hydrogen atoms were inserted in calculated positions. Least square refinements, with anisotropic thermal motion parameters for all the nonhydrogen atoms and isotropic for the remaining atoms, were employed. These data are available free of charge from the Cambridge Crystallographic Data Centre (Cambridge, Cambs, U.K.). Crystal data of complexes **1**–**6** are listed in Table 5.

### 3.3. Solution Chemistry

The stability of the zinc (II) complexes **1**–**6** was analyzed by UV-vis absorption spectra under physiological conditions. Stock concentrations of complexes **1**–**6** prepared with DMSO were diluted with PBS (pH = 7.4), and stability analysis was performed at 0, 24, 48 and 72 h at 37 °C.

### 3.4. Cell Study

In this assay, human esophageal cancer cell line (Eca-109), human lung cancer cell line (A549), human hepatocellular carcinoma cell line (Bel-7402) and mouse monocyte macrophage (RAW264.7) were used to evaluate the antiproliferative activities or cytotoxicity in vitro by CCK-8 assay for determining cell viability with cisplatin as the positive control. All the cells are purchased from the American Type Culture Collection (Manassas, VA, USA). The cells were cultured in 100 µL complete DMEM growth media containing 10% FBS and 1% PS. First, 3000 cells were added to 96-well plates and were cultured overnight in a humidified atmosphere at 37 °C with 5% CO_2_. Then, complexes **1**–**6** and cisplatin were diluted with complete DMEM growth media to obtain the working concentration, which was added to the tissue culture plate and incubated for 72 h. The CCK-8 solution was added to each well. After 3 h incubation, the absorbance of each well at 490 nm was measured using an enzyme labeling instrument (Tecan, Grödig, Salzburg, Austrla). Cell morphology was measured and imaged by an inverted microscope (Nikon eclipses TS100, Tokyo, Japan) and a Nikon digital camera (Tokyo, Japan). The 50% inhibitive concentration (IC_50_) was calculated by Graph Pad Prism 5.0 (San Diego, CA, USA).

### 3.5. Protein Binding Studies

Bovine serum albumin (BSA) was dissolved in a Tris-HCl buffer (pH = 7.4) solution to prepare a 1.6 × 10^−6^ M BSA solution. The protein concentration was measured by spectrophotometry at 280 nm with the extinction coefficient of 44,300 M^−1^ cm^−1^. Stock solutions of complexes **1**–**6** (1 × 10^−2^ M) were prepared in DMSO. The emission spectrum (300–600 nm) of BSA was monitored at the excitation wavelength of 280 nm.

### 3.6. DNA Binding Studies

#### 3.6.1. UV-Vis Spectra Titration

The UV absorption of CT-DNA that gave the ratio at 260/280 nm was 1.92, indicating that the DNA contained no protein. The molar extinction coefficient of CT-DNA was 6600 M^−1^ cm^−1^. Stock concentrations of complexes **1**–**6** prepared with DMSO were diluted with Tris-HCl (pH = 7.2) buffer, and then incubated with different concentrations of CT-DNA. Absorption spectra of the complexes incubated with different concentrations of CT-DNA (5 min) were recorded at 200–800 nm.

#### 3.6.2. Circular Dichroism (CD) Measurements

CD spectra of compounds **1**–**6** with different concentrations for interacting with CT-DNA (400 μM) were detected from 225 to 600 nm at 30 °C. Complexes **1**–**6** were dissolved in a DNA-buffer (DNA dissolved in Tris-HCl, pH = 7.2) system containing 0.4% DMSO, incubating for 24 h. 

### 3.7. Molecular Docking

The interaction between the complexes and the DNA was simulated by the molecular docking technique and the docking study was carried out with the AutoDock4.2 program (San Diego, CA, USA). The crystal data of DNA (ID: 4JD8) and BSA (ID: 3V03) were obtained from the Protein Data Bank (PDB) and the PDF format of the zinc (II) complexes was acquired from the structures of their crystals for blind docking and the output for further analysis.

## 4. Conclusions

Six new zinc (II) complexes were prepared by the reaction of ZnBr_2_ or ZnI_2_ with 4′-(substituted-phenyl)-2,2′:6′,2′′-terpyridine, which were characterized by elemental analysis, FT-IR, NMR and single crystal X-ray diffraction. The complexes exhibit interesting photoluminescence at room temperature and solution stability under physiological conditions. The antiproliferative properties were monitored using a CCK-8 assay, and the results indicate that the complexes show higher activities against Eca-109, A549 and Bel-7402 cell lines than cisplatin and display low cytotoxicity towards the normal cell line RAW264.7. In particular, complexes **1**–**2** display remarkable effects in all the cells. It is interesting that the sequence of antiproliferative activity of these compounds is consistent with the electronegativity of their substituents. Interaction of these complexes with CT-DNA and BSA was obtained by UV-Vis, CD spectra and fluorescence spectroscopy. The results show that these zinc (II) complexes interact with CT-DNA through an intercalative binding mode and have a strong binding affinity to BSA through a static quenching mechanism. The docking of the complexes with the CT-DNA fragment and BSA were simulated using molecular docking software. It is further validated that the complexes bind with DNA through intercalative binding and that these complexes spontaneously bind to the active domain of BSA, suggesting that the complexes may be candidates as potential BSA fluorophores. As seen by these results, electronic effect, electronegativity and steric effects of the substituents and anions of **1**–**6** play important roles in bioactivity, which are significant for the design of new zinc complexes with anticancer potential, and the compounds might be candidates as antitumor drugs. 

## Figures and Tables

**Table 1:** IC_50_ of complexes against human cancer cells in vitro.

Compound	Eca-109		A549		Bel-7042	
	IC_50_ (μM)	95% Confidence	IC_50_ (μM)	95% Confidence	IC_50_ (μM)	95% Confidence
		Intervals (μM)		Intervals (μM)		Intervals (μM)
**1**	0.079	0.0533–0.1188	0.309	0.6419–0.8715	0.358	0.3468–0.7850
**2**	0.066	0.0464–0.0946	0.290	0.3822–0.6072	0.331	0.2434–0.5271
**3**	0.169	0.1246–0.2311	0.482	0.2465–0.3824	0.414	0.2817–0.6089
**4**	0.075	0.0503–0.1144	0.307	0.2501–0.3819	0.397	0.2408–0.6553
**5**	0.197	0.1449–0.2693	0.748	0.2072–0.4068	0.616	0.3681–1.032
**6**	0.146	0.1208–0.1780	0.945	0.6751–0.9521	0.522	0.2533–0.4325
cisplatin	5.465	4.982–5.994	11.99	10.79–13.32	3.088	2.856–3.340

**Table 2:** Quenching constants and binding constants of compounds **1**–**6** with BSA.

Compound	*K_sv_* (M^−1^)	*K_q_* (M^−1^)	*K_b_* (M^−1^)	*n*	*R^a^*
**1**	1.76 × 10^5^	1.76 × 10^13^	4.47 × 10^3^	0.72	0.9783
**2**	1.76 × 10^5^	1.76 × 10^13^	1.80 × 10^4^	0.83	0.9989
**3**	1.15 × 10^5^	1.15 × 10^13^	2.95 × 10^5^	1.07	0.9919
**4**	1.63 × 10^5^	1.63 × 10^13^	3.80 × 10^5^	1.08	0.9943
**5**	4.63 × 10^4^	4.63 × 10^12^	1.07 × 10^4^	0.87	0.9946
**6**	7.67 × 10^4^	7.67 × 10^12^	2.24 × 10^4^	0.89	0.9960

The *R^a^* is the linear correlated coefficient for the *K_sv_* values.

**Table 3:** Docking energy of compounds **1**–**6** to DNA. Quenching constants and binding constants of compounds **1**–**6** to BSA.

Compound	BSA *n*	DNA(4JD8)
**1**	−7.69	−7.90
**2**	−8.28	−8.65
**3**	−7.00	−8.42
**4**	−6.99	−8.76
**5**	−7.26	−8.51
**6**	−7.79	−8.78

**Table 4:** Binding constants of compounds **1**–**6** with CT-DNA.

Compound	*K_b_* (M^−1^) *n*	*R^a^*
**1**	2.79 × 10^3^	0.9940
**2**	2.47 × 10^3^	0.9906
**3**	1.90 × 10^4^	0.9900
**4**	2.15 × 10^4^	0.9924
**5**	6.63 × 10^4^	0.9931
**6**	6.69 × 10^4^	0.9974

**Table 5:** Crystal data for compounds **1**–**6**.

Compound	1	2	3	4	5	6
Empirical formula	C_44_H_38_Br_4_N_6_O_6_S_2_Zn_2_	C_24_H_23_I_2_N_3_O_3_S_2_Zn	C_22_H_17_Br_2_N_3_OZn	C_22_H_17_I_2_N_3_OZn	C_22_H_17_Br_2_N_3_Zn	C_22_H_17_I_2_N_3_Zn
Formula weight	1261.3	784.74	564.58	658.56	548.58	642.56
Temperature	293(2) K	293(2) K	293(2) K	571(2) K	571(2) K	571(2) K
Crystal system	Monoclinic	Orthorhombic	Monoclinic	Monoclinic	Monoclinic	Monoclinic
space group	*C*2*/c*	*P*2_1_2_1_2_1_	*P*2_1_*/c*	*P*2_1_*/n*	*P*2_1_*/c*	*P*2_1_*/c*
*a* (Ǻ)	13.2530(5)	11.748(2)	8.2757(2)	8.8978(2)	8.23930(10)	8.1062(2)
*b* (Ǻ)	14.4141(5)	20.209(3)	14.8951(5)	15.9409(3)	14.9859(2)	15.5081(3)
*c* (Ǻ)	24.6133(9)	23.075(4)	17.1200(6)	15.8601(3)	16.7323(3)	17.3032(4)
*α* (°)	90	90	90	90	90	90
*β* (°)	95.604(3)	90	98.939(2)	105.1540(10)	97.2180(10)	96.9060(10)
*γ* (°)	90	90	90	90	90	90
Volume (Ǻ^3^)	4679.4(3)	5478.7(16)	2084.71(11)	2171.35(8)	2049.62(5)	2159.43(8)
*Z*	4	8	4	4	4	4
Calculated density (Mg/m^3^)	1.79	1.903	1.799	2.015	1.778	1.976
Absorption coefficient (mm^−1^)	4.584	3.334	5.03	3.993	5.11	4.009
*F* (000)	2496	3040	1112	1256	1080	1224
Crystal size (mm^−1^)	0.36 × 0.30 × 0.22	0.49 × 0.11 × 0.09	0.39 × 0.36 × 0.30	0.48 × 0.43 × 0.40	0.39 × 0.30 × 0.21	0.26 × 0.25 × 0.18
*θ*max, *θ*min (°)	2.95, 27.50	2.80, 29.69	2.73, 27.15	1.84, 27.12	5.52, 26.63	2.37, 27.19
Index range *h*	−16→17	−16→17	−10→10	−11→11	−9→10	−10→10
*k*	−18→15	−27→27	−19→15	−19→20	−13→18	−19→17
*l*	−31→30	−31→29	−14→21	−20→20	−20→21	−22→18
Reflections collected/unique	13958/5198	74225/14185	14640/4523	29741/4806	9325/4150	18433/4788
	[*R*(int) = 0.0236]	[*R*(int) = 0.0297]	[*R*(int) = 0.0309]	[*R*(int) = 0.0264]	[*R*(int) = 0.0541]	[*R*(int) = 0.0251]
Data/restraints/parameters	5198/0/290	14185/0/631	4523/0/262	4806/0/262	4150/0/253	4788/0/253
Goodness-of-fit on *F*2	1.012	1.021	1.017	1.013	0.943	0.994
Final *R* indices [*I* > 2*σ*(*I*)]	*R*1 = 0.0320	*R*1 = 0.0317	*R*1 = 0.0280	*R*1 = 0.0250	*R*1 = 0.0344	*R*1 = 0.0309
	*wR*2 = 0.0889	*wR*2 = 0.0777	*wR*2 = 0.0657	*wR*2 = 0.0650	*wR*2 = 0.0630	*wR*2 = 0.0825
*R* indices (all data)	*R*1 = 0.0386	*R*1 = 0.0427	*R*1 = 0.0445	*R*1 = 0.0294	*R*1 = 0.0602	*R*1 = 0.0409
	*wR*2 = 0.0931	*wR*2 = 0.0852	*wR*2 = 0.0716	*wR*2 = 0.0677	*wR*2 = 0.0660	*wR*2 = 0.0880
Largest diff. peak and hole (*e* Ǻ^−3^)	0.939 and −0.562	1.580 and −0.806	0.703 and −0.448	0.395 and −0.920	0.965 and −0.565	0.807 and −0.822
CCDC number	1969820	1969821	1969822	1969823	1969824	1969825

## Supplementary Materials

The following are available online, Figures S1–S6: 1H-NMR spectra of compounds **1**–**6** in DMSO, Figures S7–S12: IR spectra of compounds **1**–**6**, Figure S13–S17: Thermal ellipsoid plot, drawn at the 50% probability level, of compounds **2**–**6** with atomic numbering scheme, Figures S18: The UV-vis spectra of complexes **2**, **4**, **5** and **6** in PBS buffer solution over a period of 72 h, Figures S19: Microscopic photographs of Eca-109 cancer cells treated with different concentrations of compound **2**, **4**, **5** and **6**, Figures S20–S21: Microscopic photographs of the A549 and Bel-7402 cancer cells treated with different concentrations of compounds **1**–**6** and control photographs, Figures S22: Fluorescence emission spectra of 1.6 μM BSA in a Tris-HCl buffer (pH = 7.2) solution with series concentration of compounds **1**, **2**, **3** and **5**, and the curves for Stern–Volmer equation are shown as the insets, Figure S23: Docking conformation of complexes **2**–**6** to BSA and amino acid residues surrounding compounds **2**–**6**. Figures S24: Absorption spectra of 20 μM of compounds **2**, **4**, **5** and **6** in a Tris-HCl buffer (pH = 7.2) solution with series concentration of CT-DNA. The plots of A0/(A − A0) versus the concentration of CT-DNA are shown as the insets. Figure S25: CD spectra of compounds **2**, **4**, **5** and **6** to CT-DNA at different concentration ratios, Figures S26: Docking conformation of complexes **2**–**6** to DNA (PDB ID: 4JD8).
