# Supplementary material for: Synthesis, Characterization, Photoluminescence, Molecular Docking and Bioactivity of Zinc (II) Compounds Based on Different Substituents
Source: Molecules. 2020 Jul 29;25(15):3459. doi: 10.3390/molecules25153459 (PMC7436059; doi:10.3390/molecules25153459)
Supplement: Supplementary file 1 [file molecules-25-03459-s001.pdf]

Supporting information

Synthesis, Characterization, Photoluminescence, Molecular Docking and Bioactivity of Zinc (II) Compounds Based on Different Substituents

Rongping Liu [1,2], Hao Yan [3], Jinzhang Jiang [1], Jiahe Li [1], Xing Liang [1], Dengfeng Yang [4], Lixia Pan [2,*], Tisan Xie [3,*] and Zhen Ma [1,*]

1 School of Chemistry and Chemical Engineering, Guangxi University, Nanning 530004, Guangxi, China; 18877571207@163.com (R.L.); jjz19941028@gmail.com (J.J.); lijiahe006@163.com (J.L.); liangxing587@126.com (X.L.)

2 National Engineering Research Center for Non-Food Biorefinery, State Key Laboratory of Non-Food Biomass and Enzyme Technology, Guangxi Academy of Sciences, Nanning 530004, Guangxi, China

3 School of Animal Science and Technology, Guangxi University, Nanning 530004, Guangxi, China; yanhao_day@163.com

4 Guangxi Key Laboratory of Marine Natural Products and Combinatorial Biosynthesis Chemistry, Guangxi Beibu Gulf Marine Research Center, Guangxi Academy of Sciences, Nanning 530004, Guangxi, China; dengfengyang@163.com

* Correspondence: panlixia@gxas.cn (L.P.); xietisan@gxu.edu.cn (T.X.); mzmz2009@sohu.com (Z.M.); Tel.: +86-0771-250-3980 (L.P.)

Academic Editors: Simona Collina and Mariarosaria Miloso

Received: 1 July 2020; Accepted: 23 July 2020; Published: date

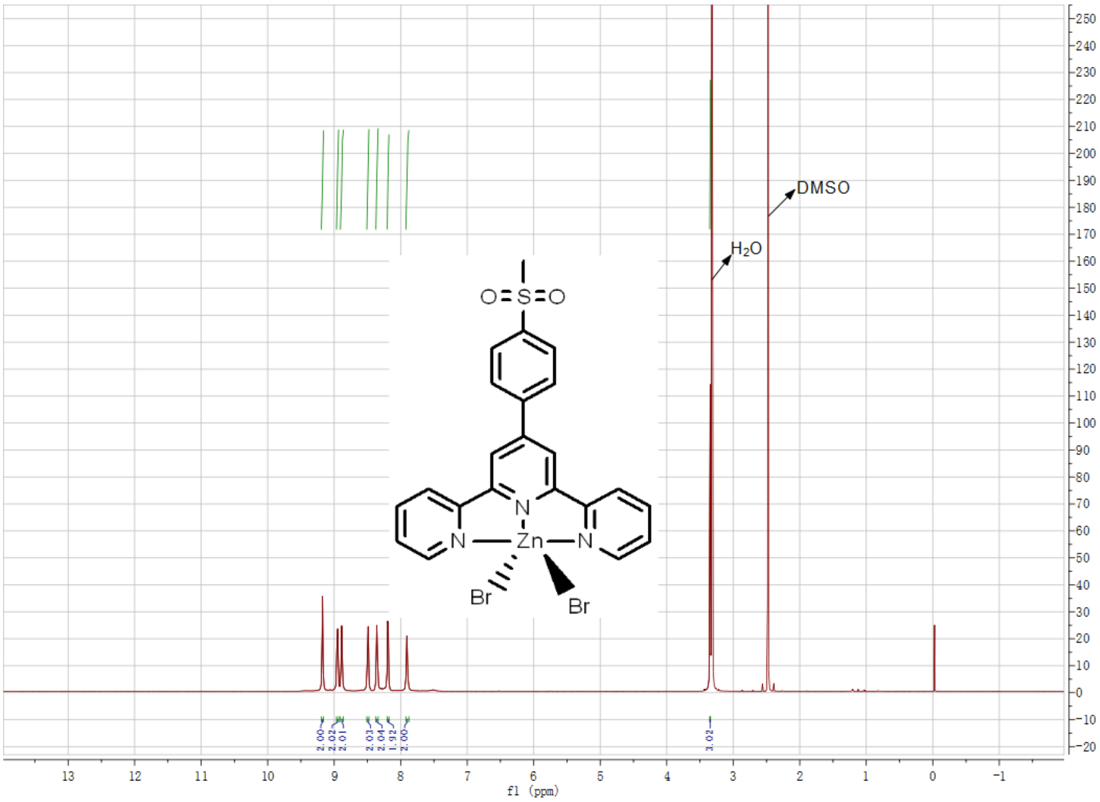

Figure S1. The ^{1}H NMR spectrum of compound **1**.

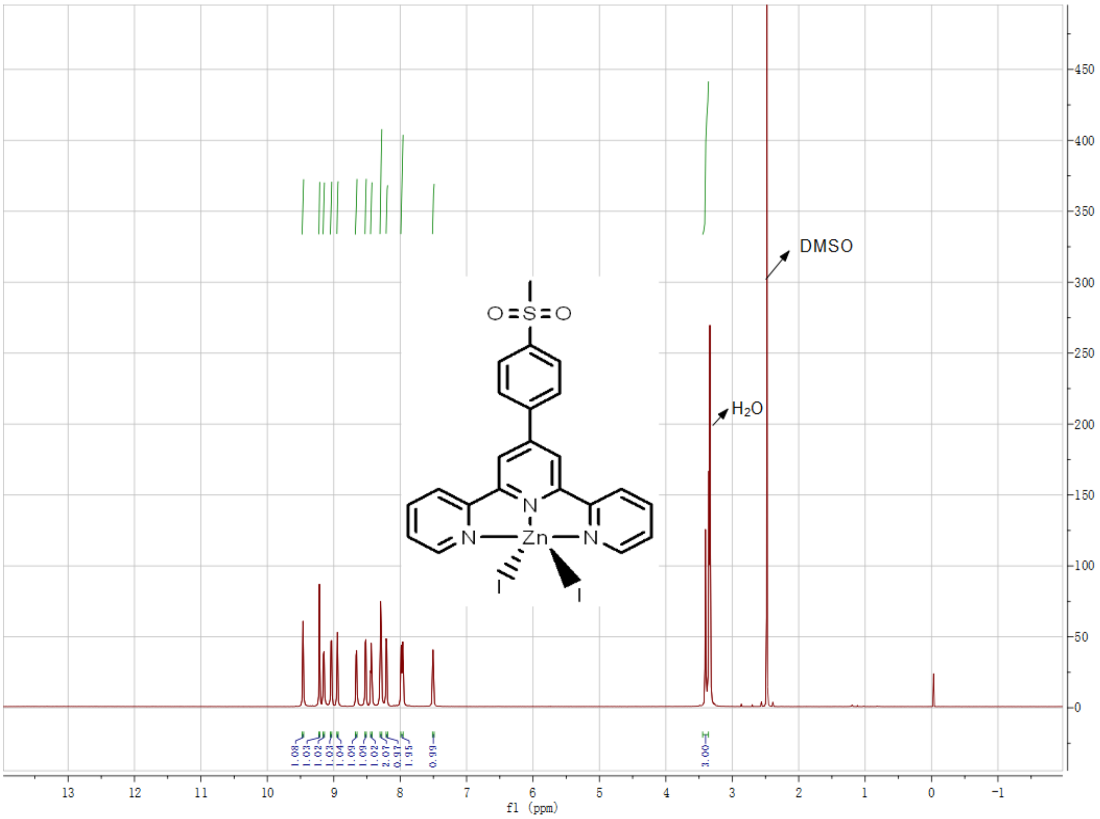

Figure S2. The ^{1}H NMR spectrum of compound **2**.

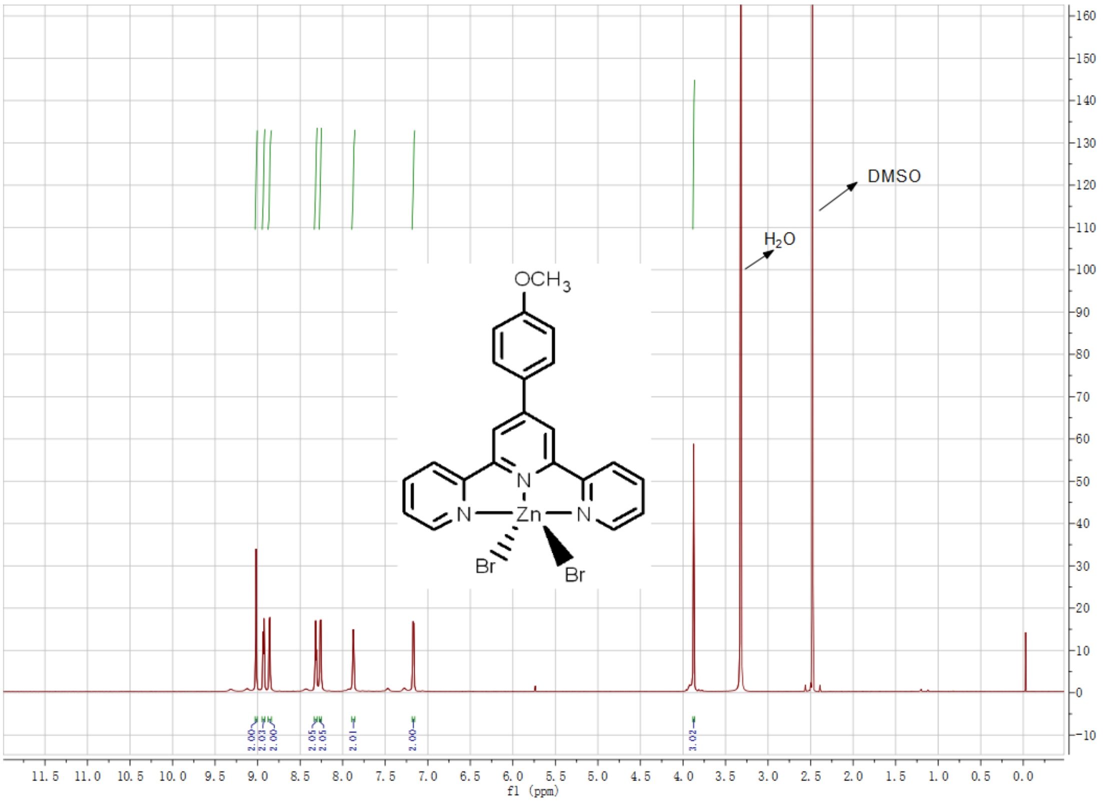

Figure S3. The ^{1}H NMR spectrum of compound **3**.

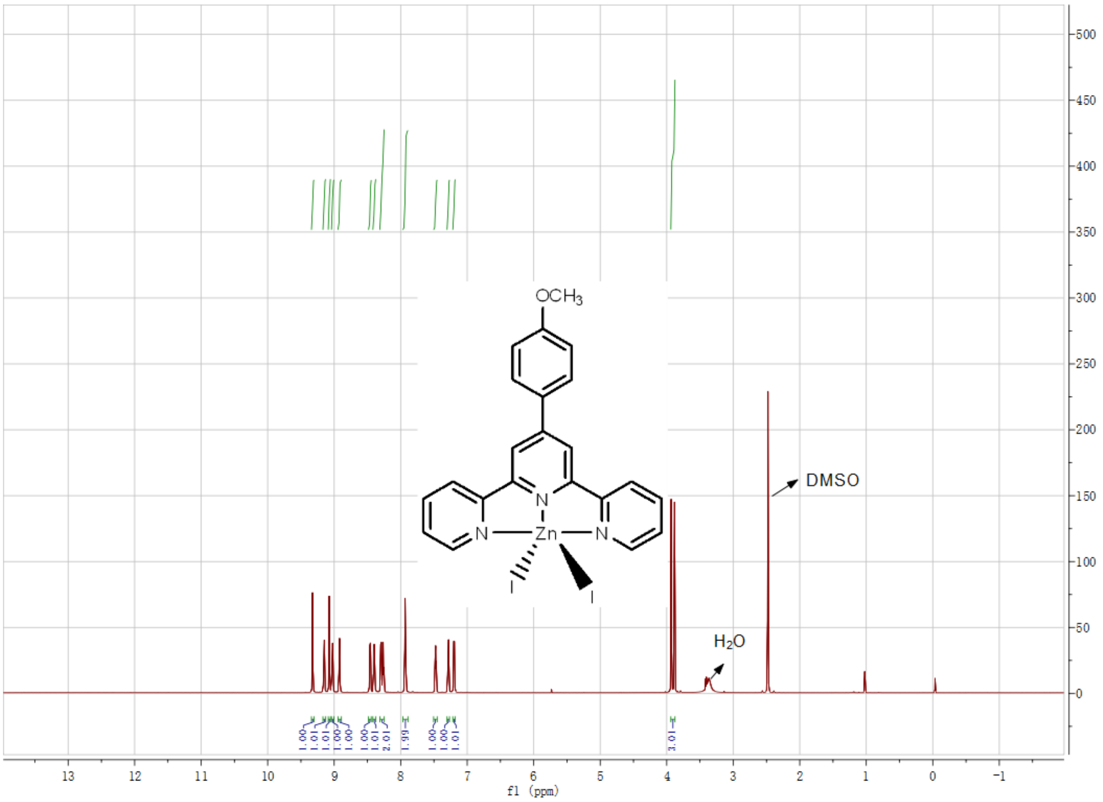

Figure S4. The ^{1}H NMR spectrum of compound **4**.

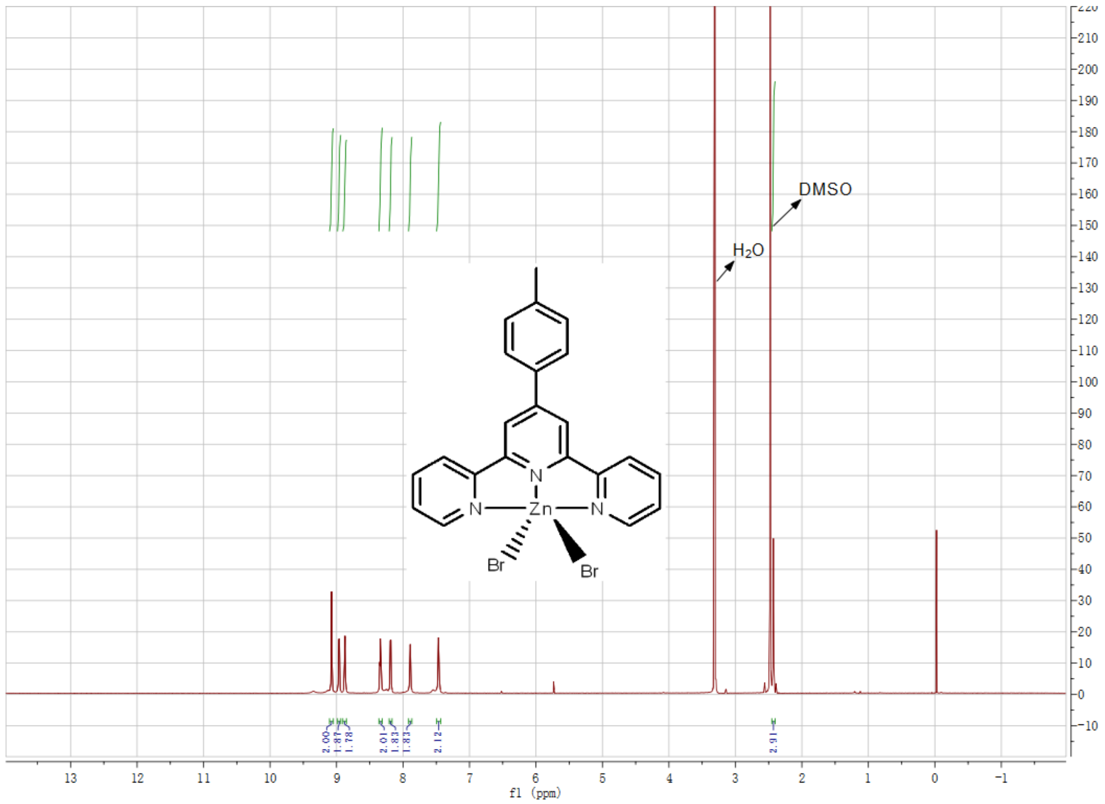

Figure S5. The ¹H NMR spectrum of compound **5.**

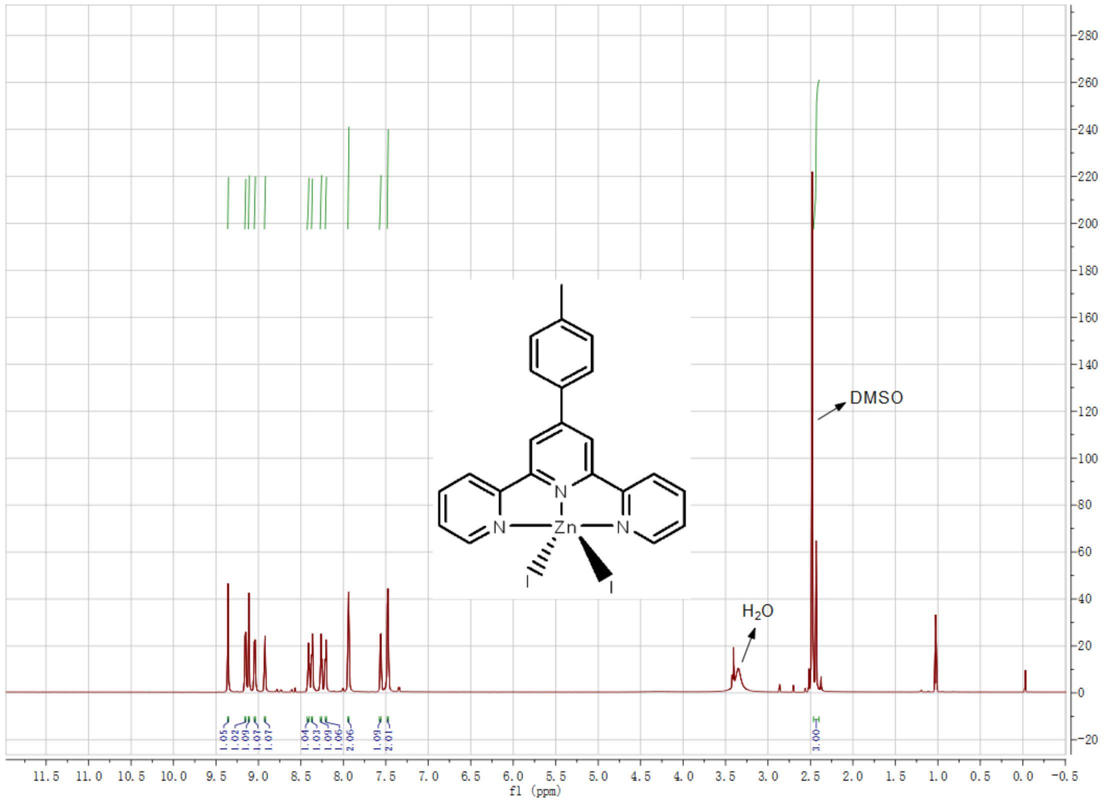

Figure S6. The ¹H NMR spectrum of compound **6.**

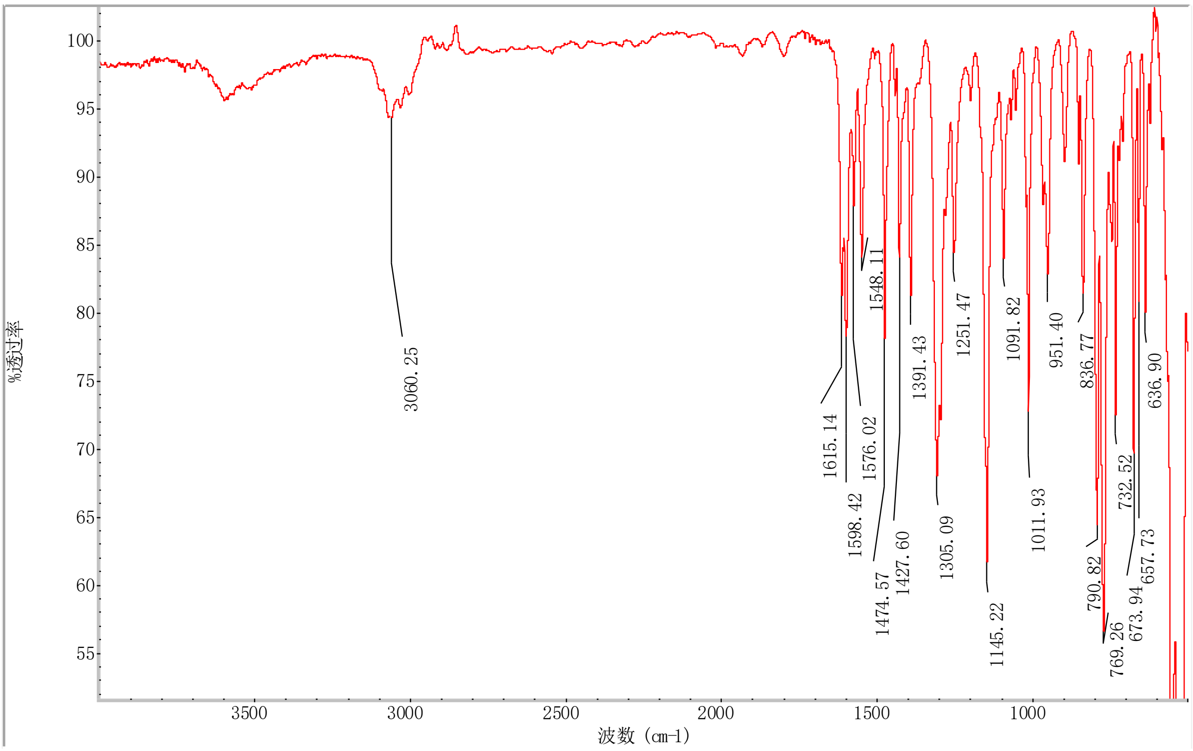

Figure S7. The IR spectrum of compound **1.**

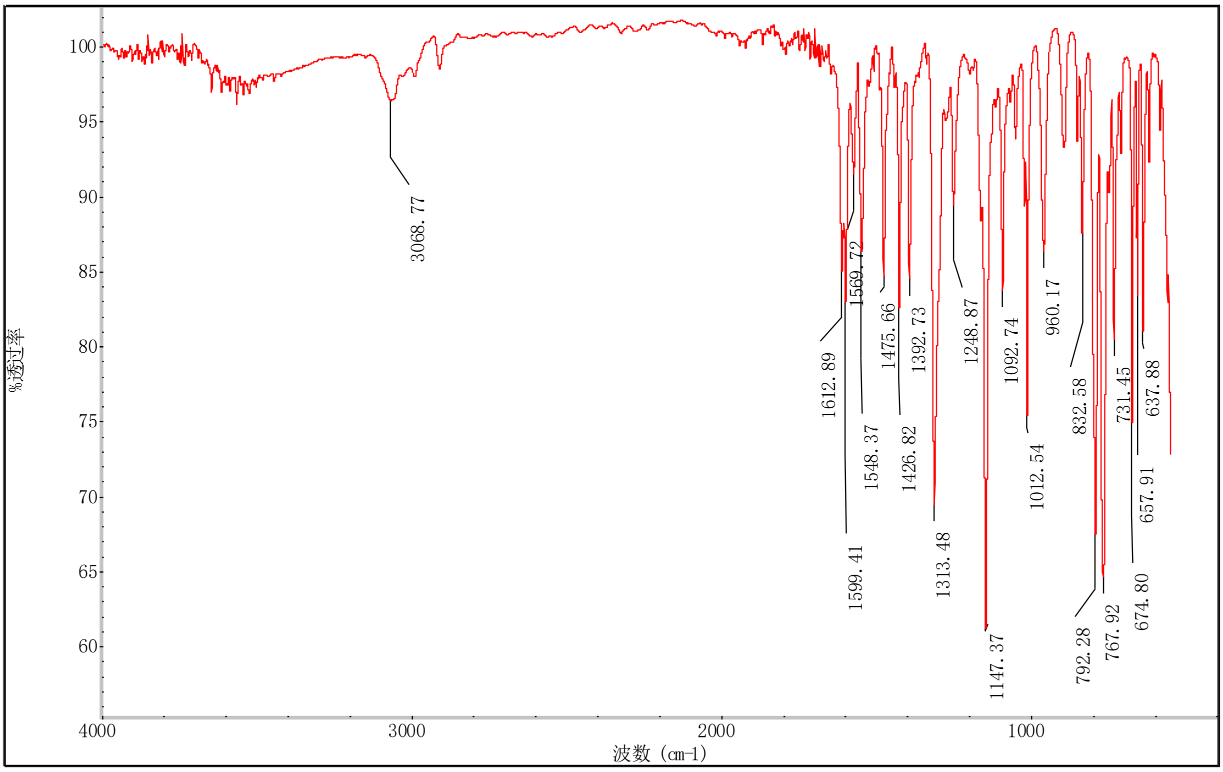

Figure S8. The IR spectrum of compound **2.**

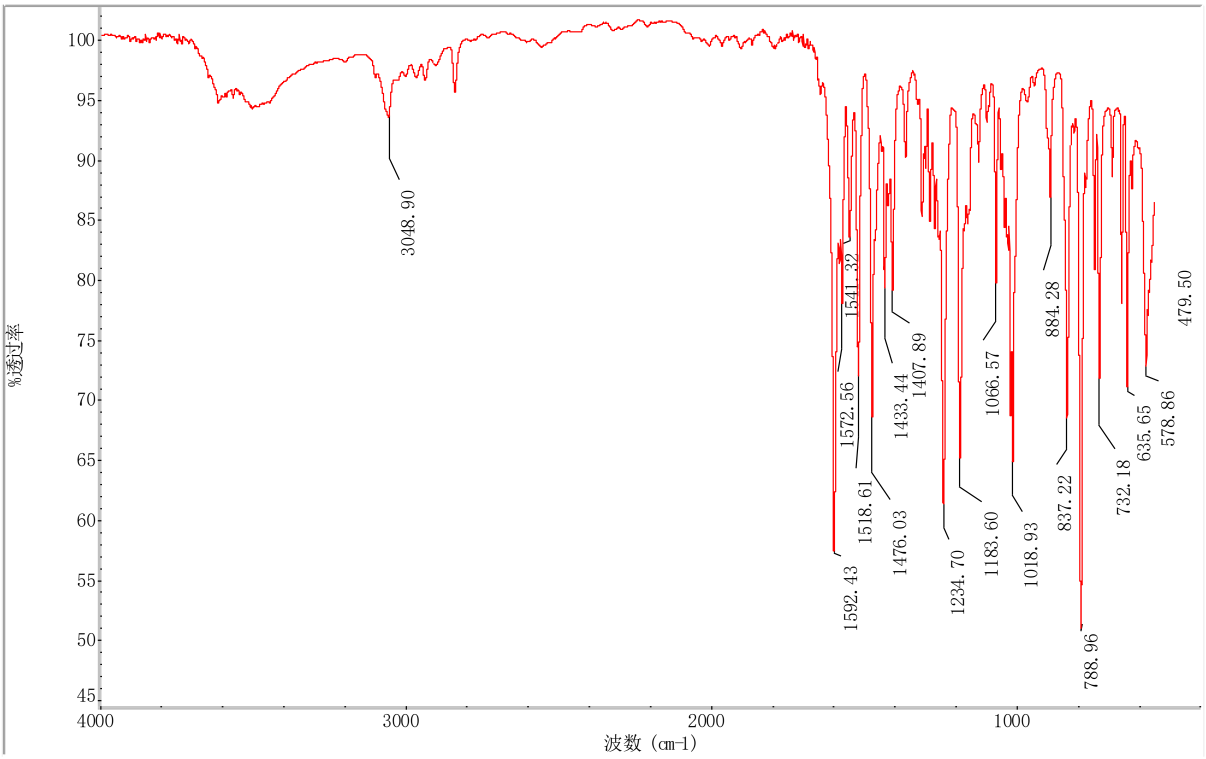

Figure S9. The IR spectrum of compound **3.**

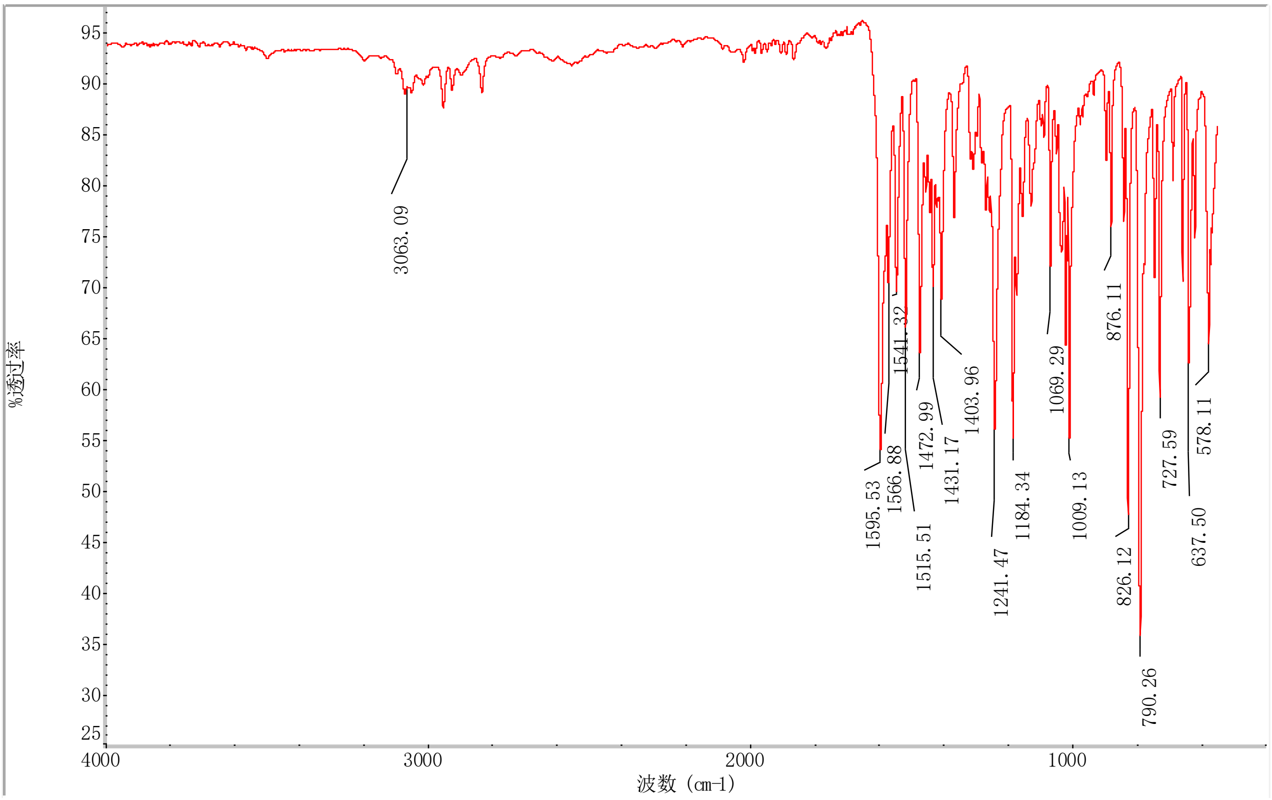

Figure S10. The IR spectrum of compound **4.**

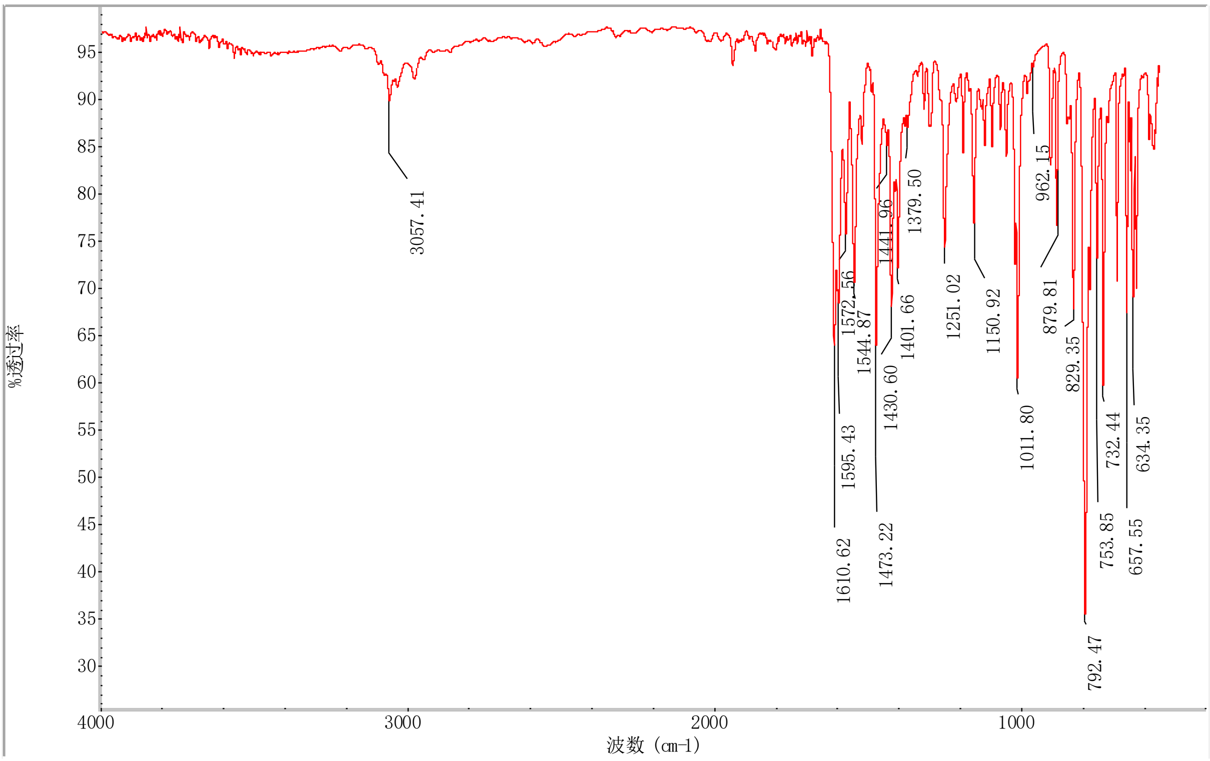

Figure S11. The IR spectrum of compound **5.**

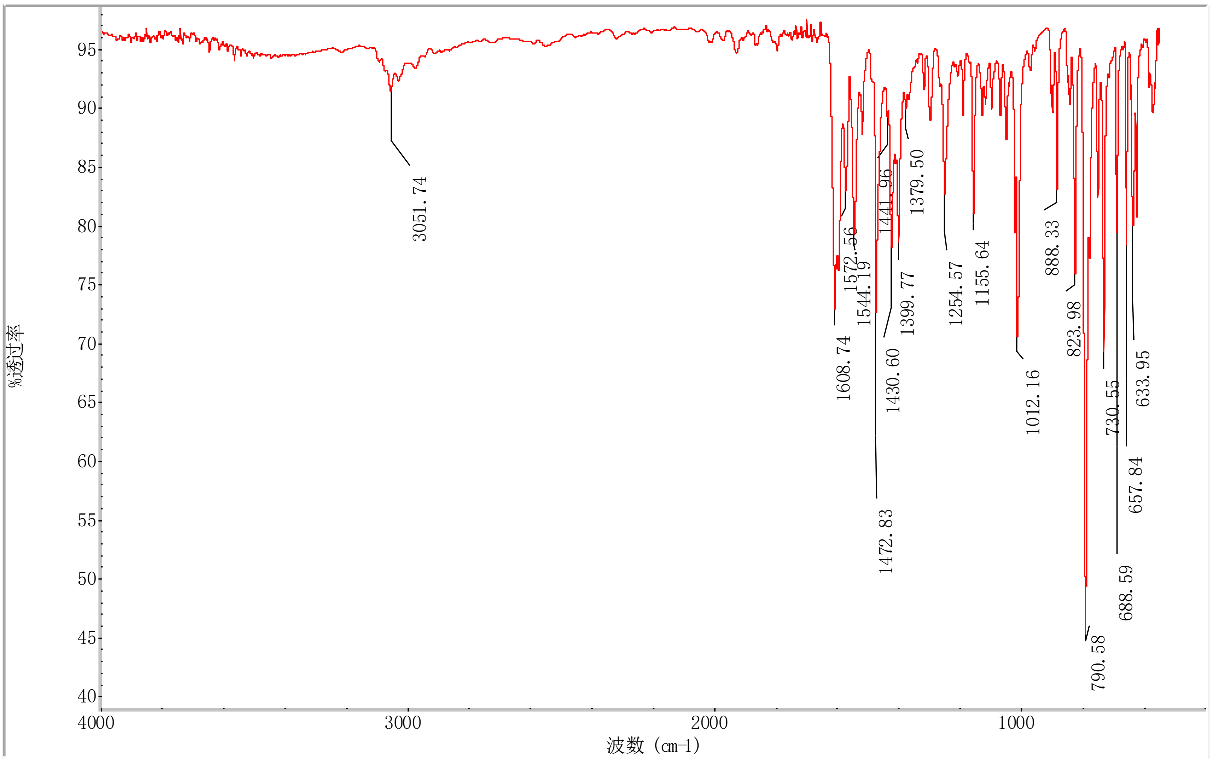

Figure S12. The IR spectrum of compound **6.**

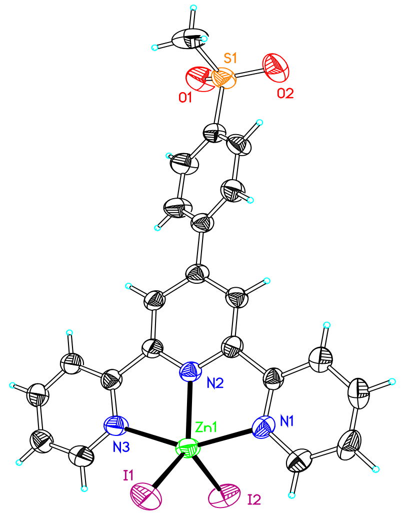

Figure S13. Thermal ellipsoid plot, drawn at the 50% probability level, of [Zn(Br)2L[1]] (1) with atomic numbering scheme. Selected bond lengths (Å) and angles (º): Zn(1)-N(2) 2.096(3), Zn(1)-N(1) 2.175(4), Zn(1)-N(3) 2.193(3), Zn(1)-I(1) 2.6147(6), Zn(1)-I(2) 2.6170(6); N(2)-Zn(1)-N(1) 74.45(12), N(2)-Zn(1)-N(3) 74.27(12), N(1)-Zn(1)-N(3) 148.71(13), N(2)-Zn(1)-I(1) 121.10(9), N(1)-Zn(1)-I(1) 95.39(10), N(3)-Zn(1)-I(1) 99.93(10), N(2)-Zn(1)-I(2) 118.99(9), N(1)-Zn(1)-I(2) 99.70(10), N(3)-Zn(1)-I(2) 96.06(9), I(1)-Zn(1)-I(2) 119.91(2).

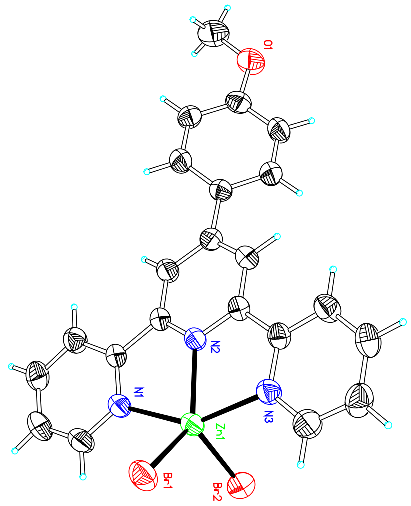

Figure S14. Thermal ellipsoid plot, drawn at the 50% probability level, of [Zn(Br)2L[1]] (1) with atomic numbering scheme. Selected bond lengths (Å) and angles (º): Zn(1)-N(2) 2.1086(19), Zn(1)-N(3) 2.209(2), Zn(1)-N(1) 2.228(2), Zn(1)-Br(2) 2.3876(4), Zn(1)-Br(1) 2.4084(4); N(2)-Zn(1)-N(3) 74.45(7), N(2)-Zn(1)-N(1) 73.80(7), N(3)-Zn(1)-N(1) 148.13(8), N(2)-Zn(1)-Br(2) 119.13(6), N(3)-Zn(1)-Br(2) 95.46(6), N(1)-Zn(1)-Br(2) 102.22(6), N(2)-Zn(1)-Br(1) 128.86(6), N(3)-Zn(1)-Br(1) 99.60(6), N(1)-Zn(1)-Br(1) 98.07(6), Br(2)-Zn(1)-Br(1) 111.967(15).

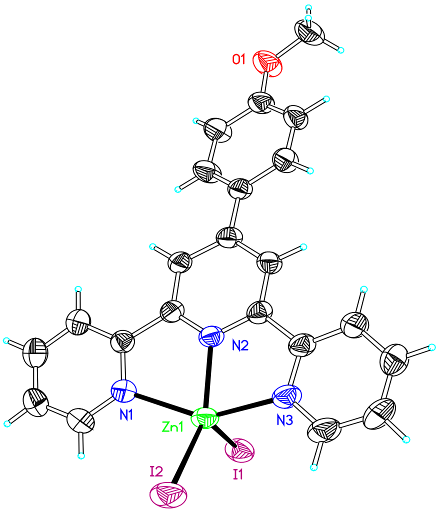

Figure S15. Thermal ellipsoid plot, drawn at the 50% probability level, of [Zn(Br)2L[1]] (1) with atomic numbering scheme. Selected bond lengths (Å) and angles (º): Zn(1)-N(2) 2.093(2), Zn(1)-N(1) 2.205(2), Zn(1)-N(3) 2.232(2), Zn(1)-I(2) 2.5919(3), Zn(1)-I(1) 2.6077(4); N(2)-Zn(1)-N(1) 74.64(8), N(2)-Zn(1)-N(3) 73.98(8), N(1)-Zn(1)-N(3) 146.64(8), N(2)-Zn(1)-I(2) 140.76(6), N(1)-Zn(1)-I(2) 97.73(6), N(3)-Zn(1)-I(2) 99.35(6), N(2)-Zn(1)-I(1) 05.09(6), N(1)-Zn(1)-I(1) 97.28(6), N(3)-Zn(1)-I(1) 101.52(7), I(2)-Zn(1)-I(1) 114.104(12).

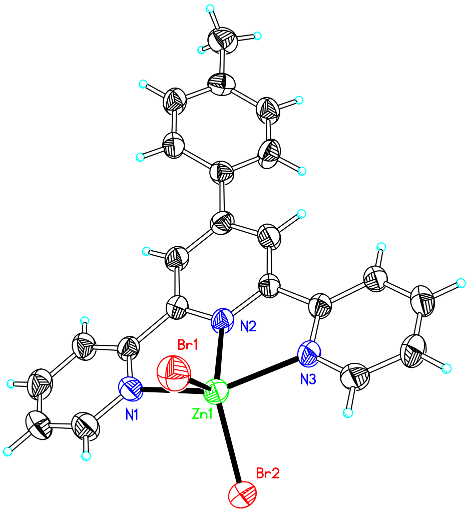

Figure S16. Thermal ellipsoid plot, drawn at the 50% probability level, of [Zn(Br)2L[1]] (1) with atomic numbering scheme. Selected bond lengths (Å) and angles (º): Zn(1)-N(2) 2.103(3), Zn(1)-N(1) 2.218(3), Zn(1)-N(3) 2.230(3), Zn(1)-Br(1) 2.3857(7), Zn(1)-Br(2) 2.4062(7); N(2)-Zn(1)-N(1) 74.35(12), N(2)-Zn(1)-N(3) 74.19(11), N(1)-Zn(1)-N(3) 148.49(11), N(2)-Zn(1)-Br(1) 119.44(9), N(1)-Zn(1)-Br(1) 97.02(9), N(3)-Zn(1)-Br(1) 99.89(9), N(2)-Zn(1)-Br(2) 125.84(9), N(1)-Zn(1)-Br(2) 98.36(9), N(3)-Zn(1)-Br(2) 98.43(8), Br(1)-Zn(1)-Br(2) 114.70(2).

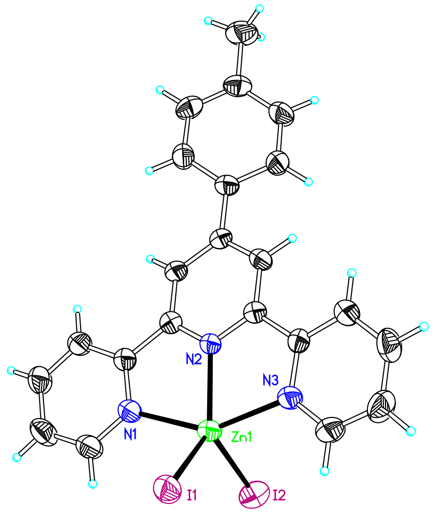

Figure S17. Thermal ellipsoid plot, drawn at the 50% probability level, of [Zn(Br)2L[1]] (1) with atomic numbering scheme. Selected bond lengths (Å) and angles (º): Zn(1)-N(2) 2.095(3), Zn(1)-N(3) 2.221(3), Zn(1)-N(1) 2.232(3), Zn(1)-I(2) 2.5838(5), Zn(1)-I(1) 2.6051(5); N(2)-Zn(1)-N(3) 74.36(11), N(2)-Zn(1)-N(1) 73.94(11), N(3)-Zn(1)-N(1) 147.98(12), N(2)-Zn(1)-I(2) 118.23(8), N(3)-Zn(1)-I(2) 97.98(9), N(1)-Zn(1)-I(2) 100.82(9), N(2)-Zn(1)-I(1) 129.08(8), N(3)-Zn(1)-I(1) 97.59(8), N(1)-Zn(1)-I(1) 98.76(8), I(2)-Zn(1)-I(1) 112.661(18).

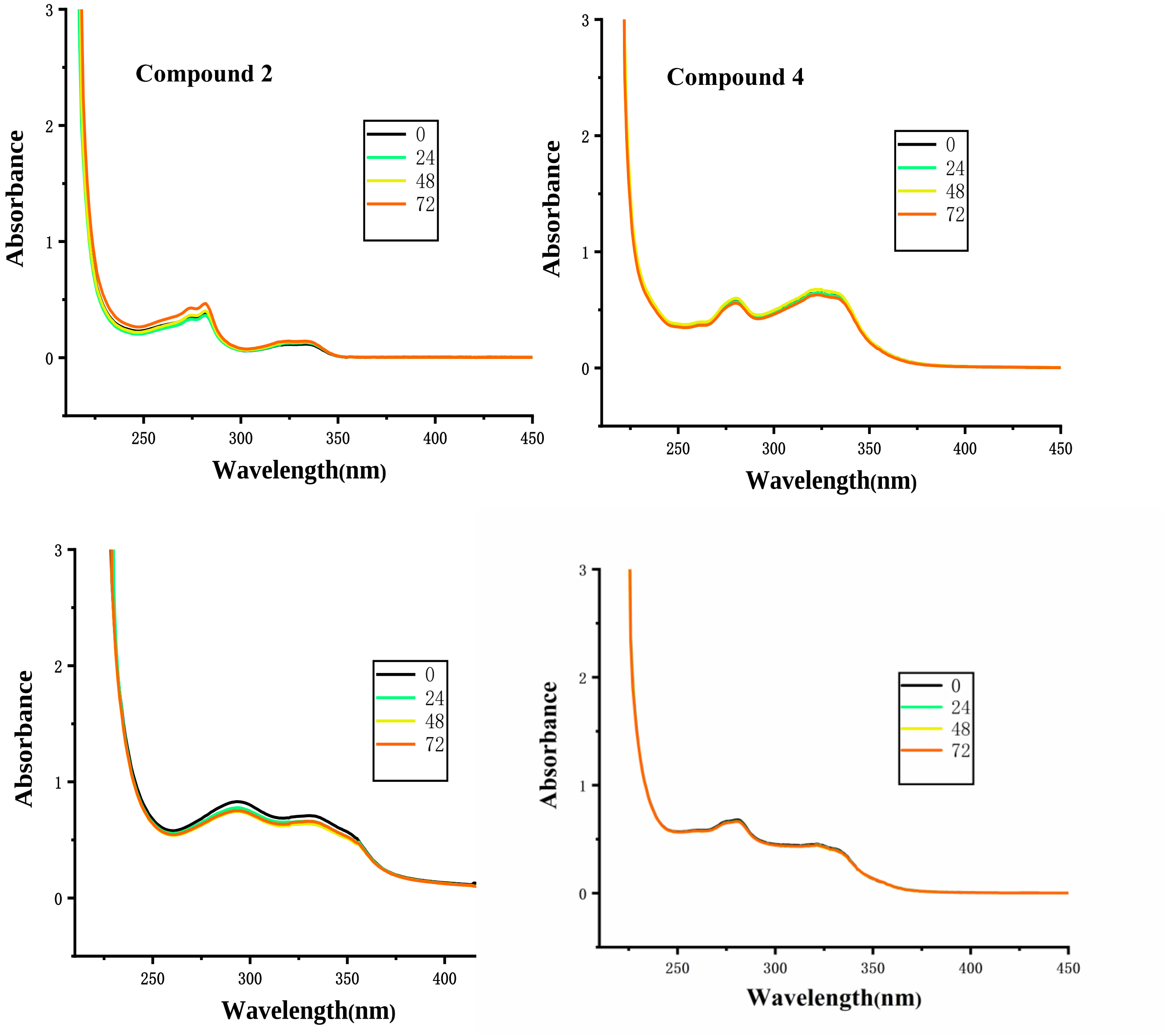

Figure S18. The UV–vis spectra of complexes **2, 4, 5** and **6** in PBS buffer solution over a period of 72*h*.

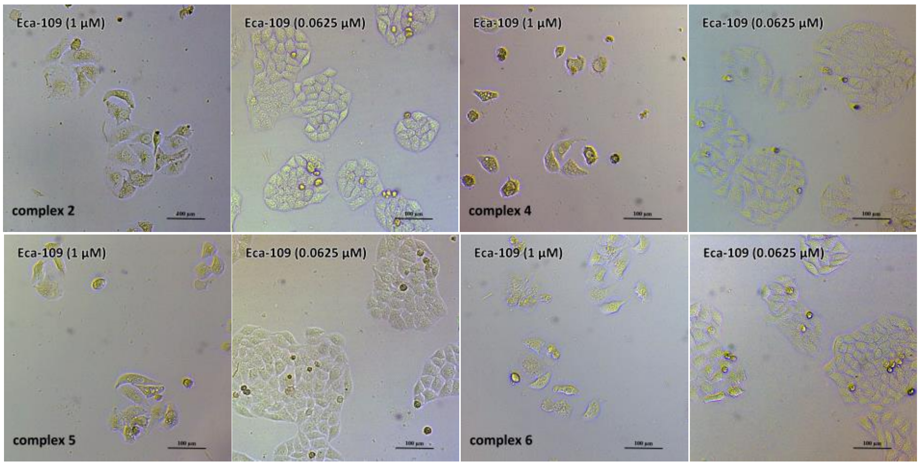

Figure S19. The microscopic photographs of Eca-109 cancer cells treated with different concentrations of compound **2, 4, 5 and 6.**

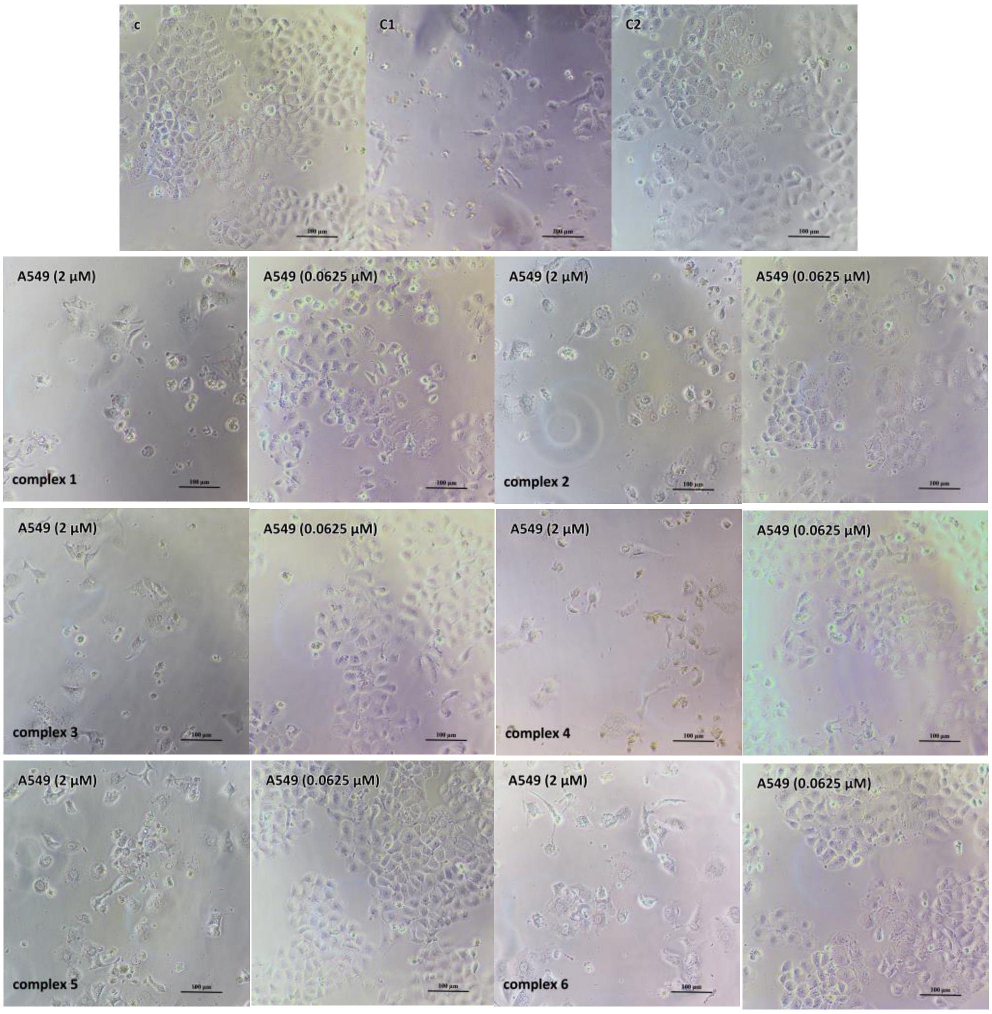

Figure S20. The microscopic photographs of A549 cancer cells treated with different concentrations of compounds and control photographs (**C** for blank group, **C1** for 5 μM cisplatin and **C2** for 40 μM cisplatin).

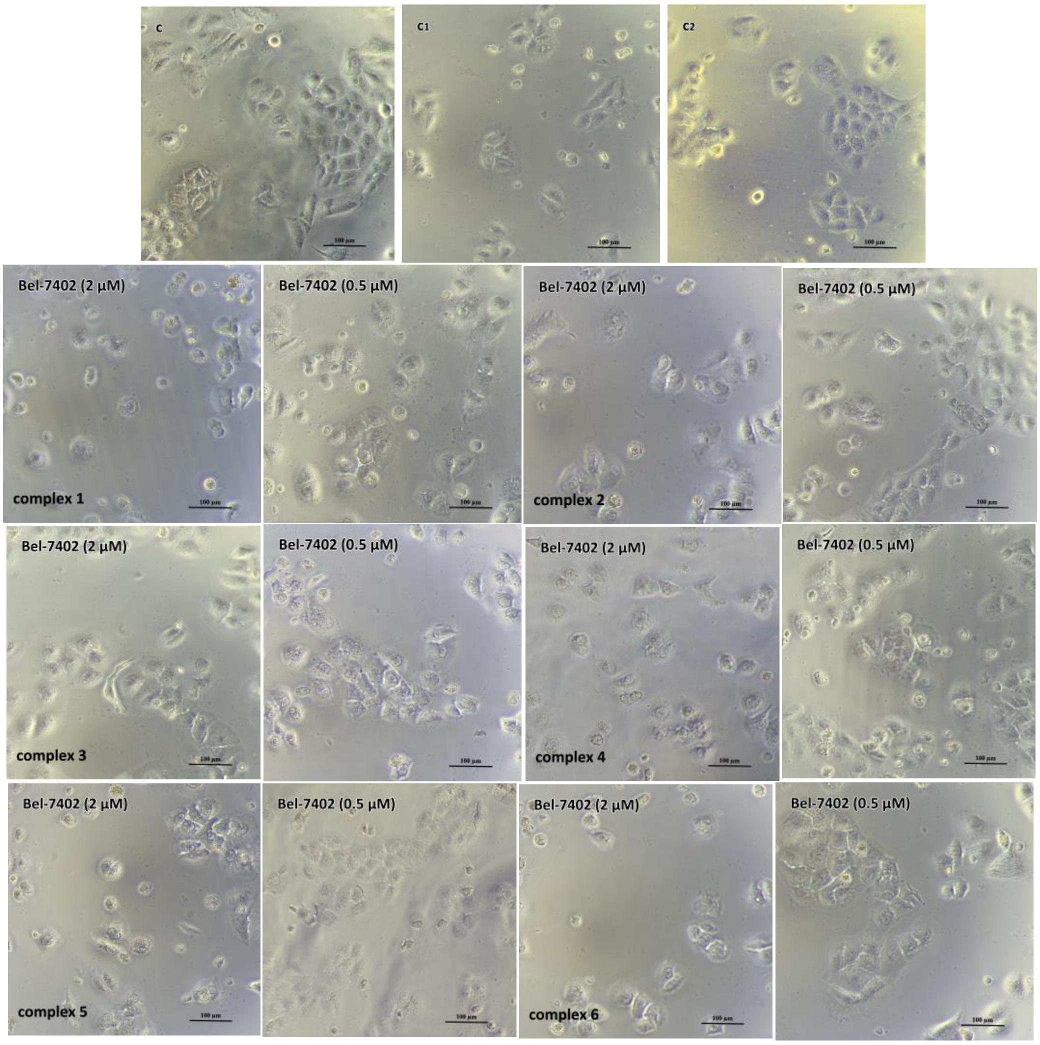

Figure S21. The microscopic photographs of Bel-7402 cancer cells treated with different concentrations of compounds and control photographs (**C** for blank group, **C1** for 2.5 μM cisplatin and **C2** for 5 μM cisplatin).

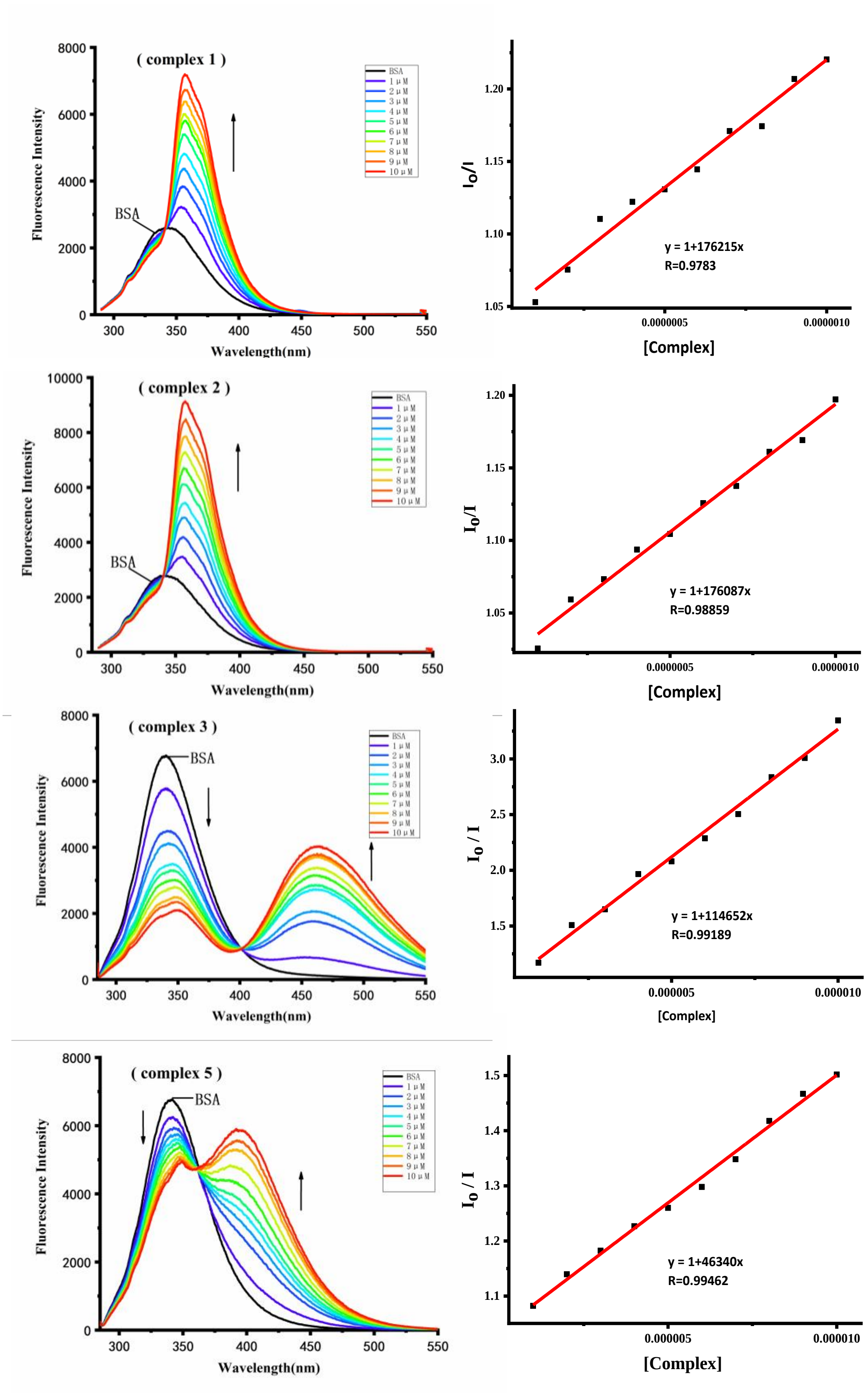

Figure S22. Fluorescence emission spectra of 1.6 μM BSA in Tris-HCl buffer (PH = 7.2) solution with series concentration of compound **1, 2, 3** and **5**, and the curves for Stern-Volmer equation as the insets.

(Compound 2)

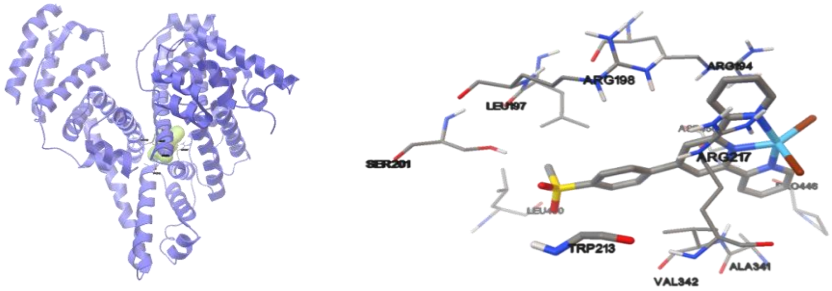

(Compound 3)

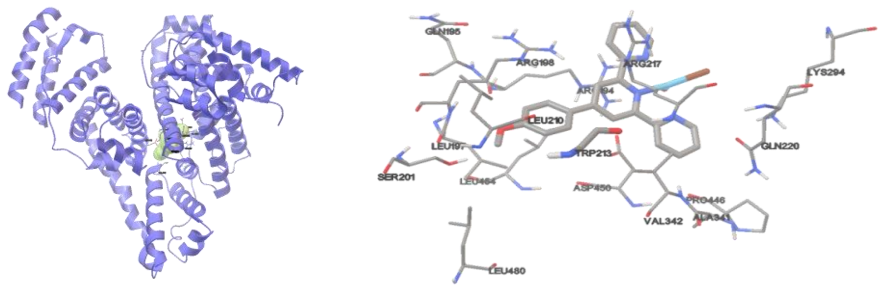

(Compound 4)

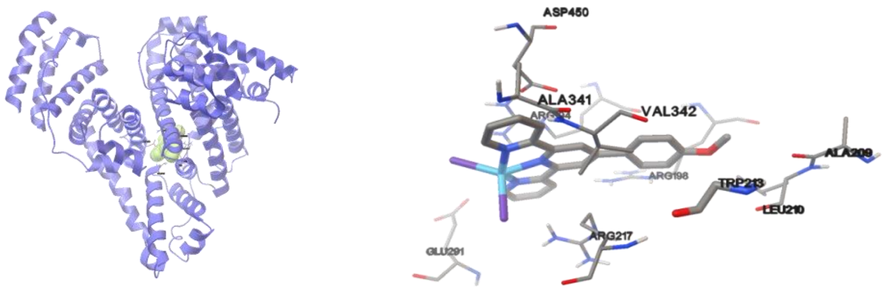

(Compound 5)

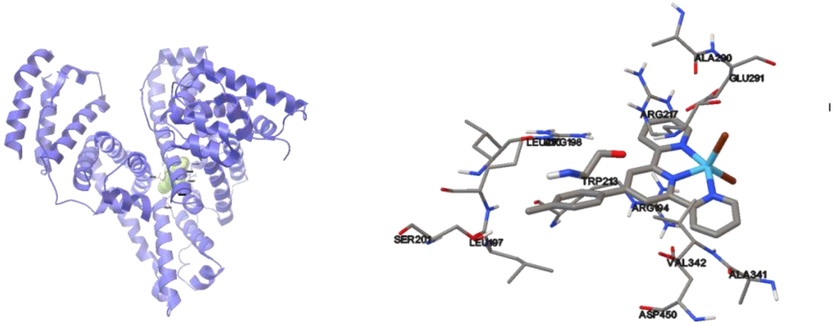

(Compound 6)

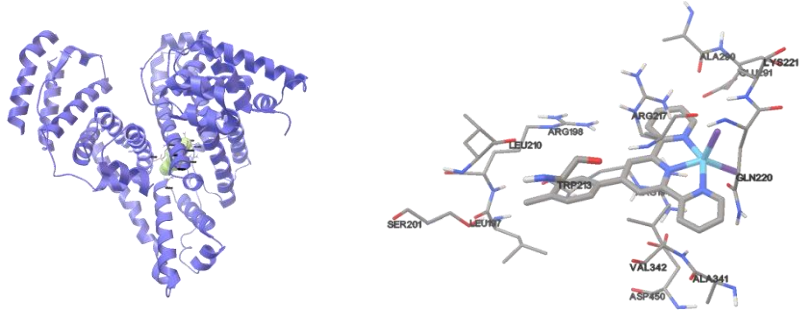

Figure S23. The docking conformation of complexes **2–6** to BSA， and amino acid residues surrounding compounds **2–6**.

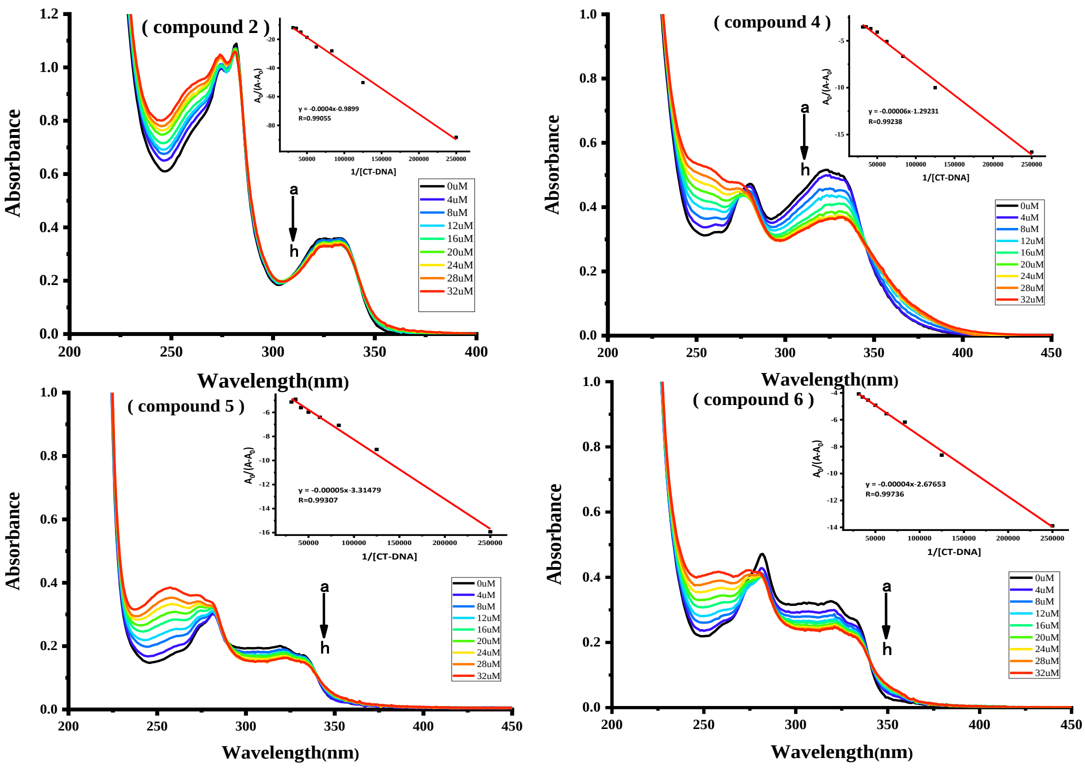

Figure S24. Absorption spectra of 20μM compounds **2, 4, 5** and **6** in Tris-HCl buffer (PH=7.2) solution with series concentration of CT-DNA, The plots of $A_0/(A–A_0)$ versus the concentration of CT-DNA as the insets.

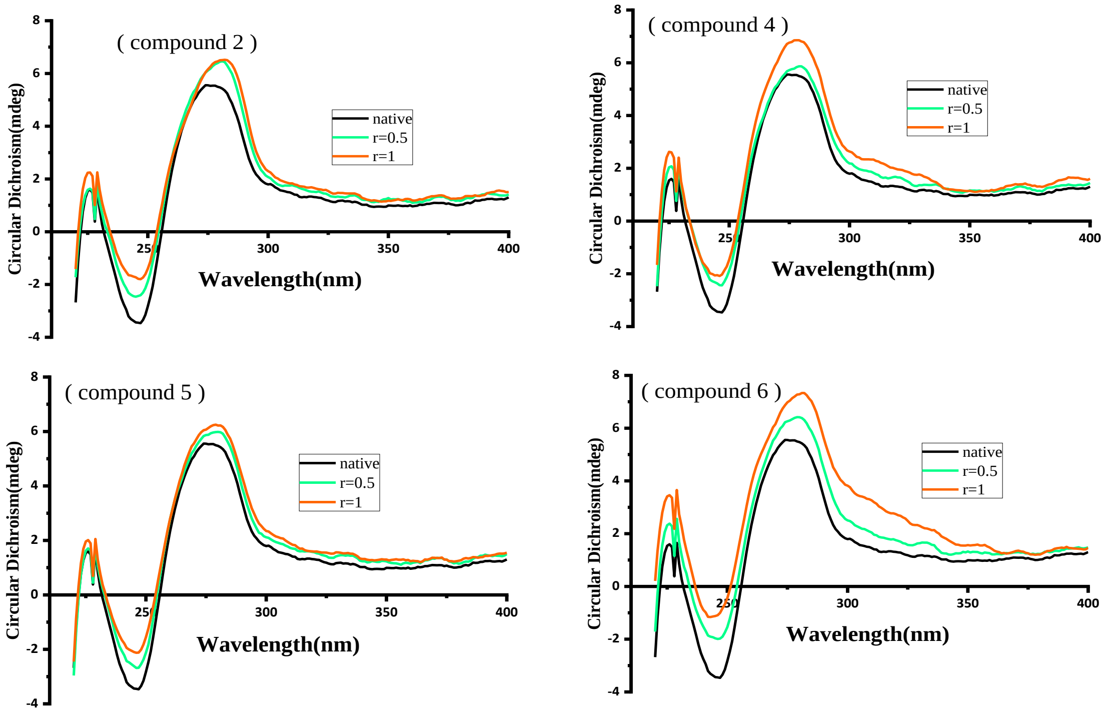

Figure S25. CD spectra of compounds **2, 4, 5** and **6** to CT-DNA at different concentration ratios.

(Compound 2)

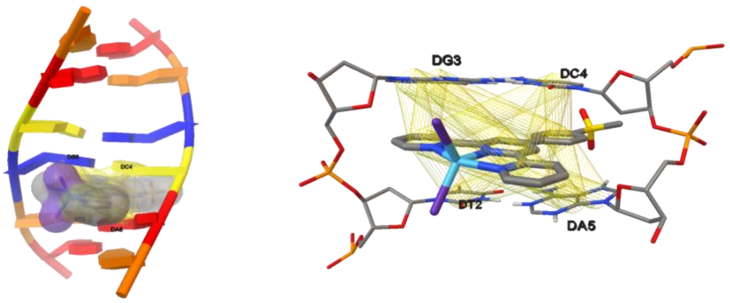

(Compound 3)

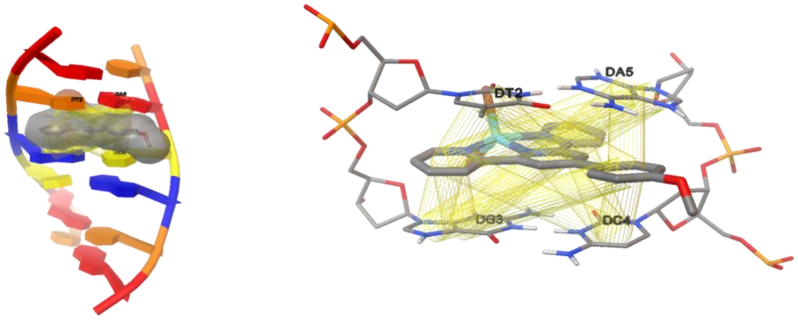

(Compound 4)

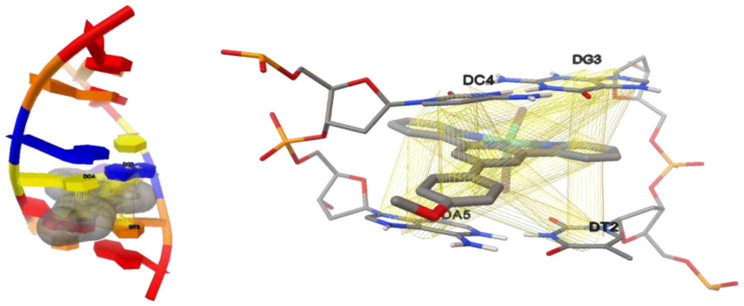

(Compound 5)

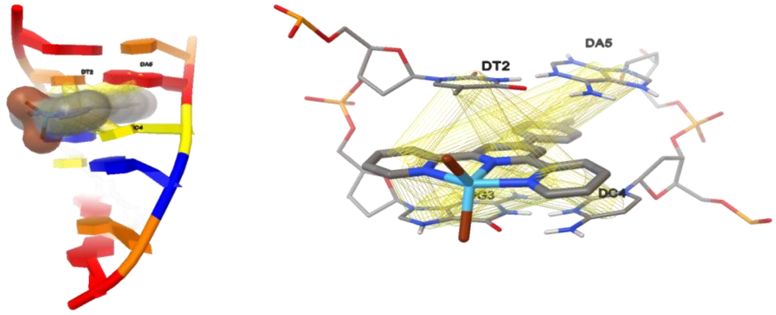

(Compound 6)

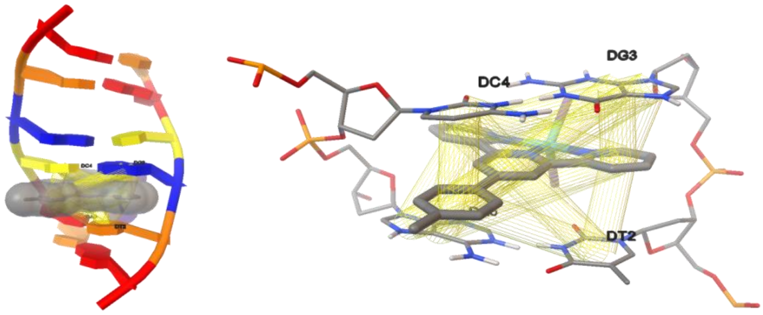

Figure S26. The docking conformation of complexes **2–6** to DNA (PDB ID: 4JD8).
